# ARHGAP18 complexes with both YAP and Merlin and is required for basal actin bundles

**DOI:** 10.1091/mbc.E26-03-0143

**Published:** 2026-06-30

**Authors:** Emma C. Murray, Gillian M. Hodge, Khanh Pham, Shane Fraher, Leighton S. Lee, Cameron A.R. Mitchell, Namya Manoj, Christine E. Schaner Tooley, Yongho Bae, Andrew T. Lombardo

**Affiliations:** ^a^Department of Biochemistry, State University of New York at Buffalo, Buffalo 14203 NY; ^b^Department of Pathology and Anatomical Sciences, State University of New York at Buffalo, Buffalo 14203 NY; ^c^Department of Molecular Biology and Genetics, Cornell University, Ithaca NY; University of Queensland

## Abstract

The organization of the cell's cytoskeletal filaments is coordinated through a complex network of signaling cascades activated by both internal and external cues. Two major actin regulatory pathways are signal transduction through Rho family GTPases and growth and proliferation signaling through the Hippo pathway. These two pathways define the actin cytoskeleton, controlling foundational cellular attributes such as morphology and the organization of actin-based structures, and are hijacked to promote proliferation and motility in aggressive cancers. In this study, we use human epithelial cells to investigate the interplay between the Hippo and Rho Family signaling pathways. We identify that the RhoA GTPase-activating protein, ARHGAP18, forms a complex with two Hippo pathway components, the tumor suppressor Merlin (NF2), and the transcriptional coactivator YAP. Using super-resolution STORM microscopy, we characterize single-filament-level changes in the actin cytoskeleton that arise from CRISPR/CAS9 knockout of ARHGAP18. We report that the loss of ARHGAP18 results in cytoskeletal alterations associated with dysregulation of RhoA signaling at apical structures and aberrant nuclear localization of YAP. These findings provide additional support for models suggesting that Hippo and Rho family GTPase signaling cascades may be temporally and spatially coordinated in the regulation of the actin cytoskeleton.

## Abstract (executive summary)

- Rho GTPases regulate actin via spatially precise signaling. ARHGAP18, a RhoA GAP, has previously been linked to HIPPO signaling, yet the mechanism and its direct link to HIPPO signaling are unclear.
- ARHGAP18 loss ablates nearly all basal actin bundles and focal adhesions in human cells. These defects can be rescued by a GAP-deficient variant, indicating a RhoA GAP-independent function. ARHGAP18 forms a complex with HIPPO signaling components YAP and Merlin through an N-terminal region outside its GAP domain.
- Future work will be required to define whether HIPPO and RhoA signaling are coordinated through ARHGAP18.

## INTRODUCTION

Extracellular signals are transduced through the cell membrane, in part through receptor ligand activation of Rho family GTPases. Rho family proteins bind and cycle between an active, guanosine triphosphate (GTP) bound state and an inactive guanosine diphosphate (GDP) bound state. This cycling has traditionally been referred to as a “molecular switch” that turns on or off major actin regulatory components (Hall and Nobes, 2000). These components include the actin-based molecular motor non-muscle myosin 2 (NM-Myo-2) through Rho-associated kinase (ROCK) or the actin severing protein cofilin, through LIM kinase (Julian and Olson, 2014). Recent understanding has advanced the “on/off” characterization of Rho GTPases to a more nuanced regulatory environment where both GTP and GDP-bound states promote molecular interactions temporally and spatially (Denk-Lobnig and Martin, 2019). Nonetheless, Rho family GTPases are well established as essential cellular signals for actin nucleation, polymerization, and organization into higher-order branched or bundled structures (Hall, 1998). These signals serve to order the cell into polarized domains, defining the shape of the cell, the morphological structures present (e.g., microvilli, focal adhesions, etc.), and the cell–cell contacts required to build tissues.

Recently, a conserved mechanism of actin regulation has emerged where domain-specific regulation by Rho effectors is controlled on a minute temporal and spatial scale (Landino *et al.*, 2021; Swider *et al.*, 2022; Sepaniac *et al.*, 2023; Jackson *et al.*, 2024). Our group recently characterized an epithelial-specific RhoA signaling pathway involving ARHGAP18 organizing at the apical surface of polarized cells on the scale of 100 nm (Lombardo *et al.*, 2024). Separately, we showed in human colorectal CaCo-2 cells that the RAB GTPase-activating protein (GAP) Epi64A localizes to an approximately 75 nm region at the base of microvilli to regulate the terminal web of actin, controlling cell shape (Miller *et al.*, 2022). Collectively, these findings indicate that from flies to humans, the cell regulates Rho family GTPases on the scale of each actin structure to build the correct structure at the correct time. In the progress of our studies, we discovered an unexpected phenotype in cells genetically lacking ARHGAP18. These cells exhibited a basal membrane actin phenotype opposite to what would be expected from the loss of a RhoA GAP, challenging the emerging model in the field.

ARHGAP18 shows a strong specificity for accelerating the hydrolysis of GTP of RhoA in epithelial cells (Maeda *et al.*, 2011) but also exhibits increased activity toward RhoC in endothelial cells (Chang *et al.*, 2014). RhoA-GTP regulates the actin cytoskeleton by promoting multiple downstream kinase pathways. Of these, two kinases are required for the formation of organized actin in polarized epithelial cells: Rho-associated protein kinase (ROCK) and the homologues Lymphocyte Oriented Kinase/ STE20-like Kinase (LOK/SLK). These kinases increase actin bundling in both stress fibers through non-muscle myosin-2 (NM-Myo-2) and microvilli through activation of the Ezrin, Radixin, Moesin (ERM) family in concert with recruitment of additional microvilli-specific components (Gaeta *et al.*, 2021; Zaman *et al.*, 2021; Morales *et al.*, 2023). RhoA GAPs limit these effects by accelerating the transition of RhoA, negatively regulating it toward its inactivated GDP-bound state. Thus, increased RhoA GAP activity canonically scales inversely with the formation of bundled actin filaments. However, ARHGAP18 knockdown results in the disorganization of stress fibers in human cells (Lay *et al.*, 2019). The fly homolog of ARHGAP18 was similarly reported to positively regulate actin bundling through Rho1 and was subsequently named conundrum (Conu) due to this unexpected phenotype (Neisch *et al.*, 2013). Astonishingly, knockdown of ARHGAP18 in human 3D spheroids was reported to reduce NM-Myo-2 activation and resulted in a loss of stress fibers (Porazinski *et al.*, 2015).

To explain these results, ARHGAP18 is hypothesized to act as a non-traditional RhoA GAP where genetic screening linked its enigmatic function to the cytoskeletal transcription factor Yes-associated protein (YAP; Porazinski *et al.*, 2015). Additionally, in ARHGAP18 deleted mice, YAP inappropriately localizes to the nucleus (Coleman *et al.*, 2020). YAP and the transcriptional co-activator with PDZ-binding motif (TAZ) are the downstream effectors of the Hippo pathway. Hippo signaling originates with mechanosensation and serum availability at apical and junctional membranes in part through the ERM-related protein Merlin (also known as neurofibromin 2 or NF2; Harvey *et al.*, 2013; Reginensi *et al.*, 2016). Hippo signaling shuttles YAP and TAZ between the cytoplasm and nucleus. When localized to the nucleus, YAP/TAZ promotes transcriptional changes associated with cell proliferation and actin polymerization. Phosphorylation of YAP at the S127 residue targets it to the cytoplasm for eventual degradation. Alternatively, the activation of YAP and TAZ is pervasive in human malignancies (Zanconato *et al.*, 2016). The combined results of the studies linking ARHGAP18 to YAP/TAZ reported both upstream and downstream regulation, indicating a possible feedback loop. However, the mechanism of this potential relationship remained unknown.

In this manuscript, we characterize alterations in actin network organization in Jeg-3 human epithelial cells lacking ARHGAP18. Using super-resolution STORM microscopy to resolve actin filament architecture at <40 nm resolution, we observe a pronounced reduction in basal actin bundles, including stress fibers and filopodia, following ARHGAP18 loss. Using a GAP-deficient ARHGAP18 variant, we find evidence suggesting that ARHGAP18 may influence actin organization through both GAP-dependent and GAP-independent mechanisms. In apical actin structures, such as microvilli, we've recently observed expected defects for the loss of a RhoA GAP consistent with overactive RhoA signaling (Lombardo *et al.* 2024). However, in basal actin structures, such as focal adhesions and stress fibers, the near-total loss of actomyosin bundles is inconsistent with enhanced local RhoA signaling. In parallel, ARHGAP18 is found to associate with the Hippo pathway components YAP and Merlin, with changes in YAP localization upon loss of ARHGAP18 correlating with cytoskeletal alterations, suggesting a potential Rho GAP-independent contribution. However, the functional consequences of the ARHGAP18 to YAP and Merlin interactions remain to be fully determined. Together, these findings are consistent with a model in which ARHGAP18 may contribute to the coordination of Hippo and Rho family signaling pathways in regulating actin cytoskeletal organization, although the precise mechanistic links underlying this coordination remain an important area for future investigation.

## RESULTS

### ARHGAP18 preferentially localizes to microvilli yet maintains a reduced presence in the cytoplasm and nucleus

**FIGURE 1:**
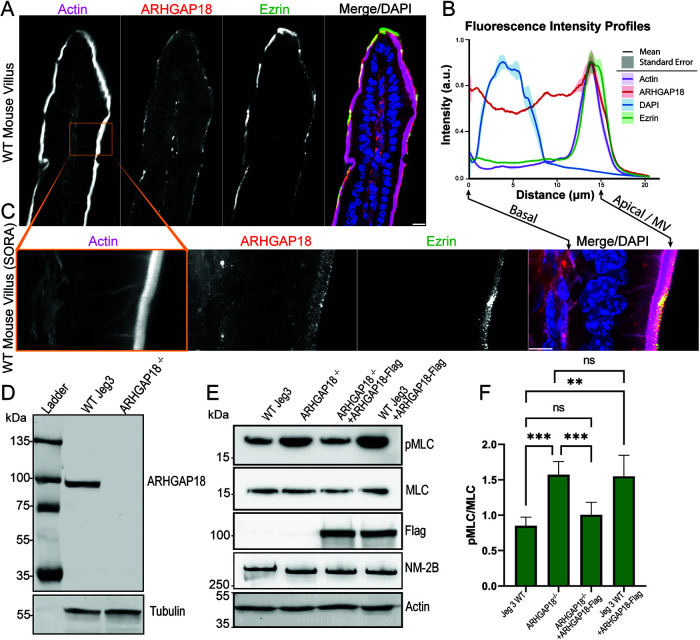
Characterization of ARHGAP18 in WT Mouse Intestinal Tissue and ARHGAP18 Knockout in Human Epithelial Cells. A) Spinning disk confocal immunofluorescent imaging of ezrin, ARHGAP18, and actin in WT mouse intestinal villi tissue. Scale bar 10 µm. B) Fluorescence intensity profiles of DAPI, anti-ARHGAP18, anti-ezrin, and phalloidin actin in WT mouse intestinal villi tissue. Intensity profiles are traced from the basal membrane to the lumen of the intestine through the nucleus of each individual cell measured. Dark lines indicate averaged intensity across all measured cells while the shaded region indicates the standard error of the mean along the profile. The DAPI peak indicates the averaged position of the nucleus while the colocalized ezrin actin peak indicate the averaged position of the microvilli with the measured intensity of ARHGAP18 relative to overlayed to indicate it's average relative abundance in each cellular structure. C) Spinning disk confocal SORA immunofluorescent imaging of DAPI, phalloidin actin, anti-ARHGAP18, and anti-ezrin in WT mouse intestinal villi tissue. Scale bar 5 µm D) Western blot of ARHGAP18 and Tubulin in WT Jeg-3 cells and ARHGAP18^-/-^ knockout (KO) cells. E) Western blot of Non-muscle Myosin 2B (NM-Myo-2B), Myosin Light Chain (MLC), anti-flag (ARHGAP18-flag), and phosphorylated MLC (pMLC) in WT and KO cells with tagged ARHGAP18 rescue or overexpression. F) Quantification of western blot intensity of the ratio of pMLC fraction over total MLC in the conditions from (E). Bars indicate Mean ± SEM; Significance by paired t-test (*n* = 6, * = *p* ≤ 0.05, ** = *p* ≤ 0.01, *** = *p* ≤ 0.001, n.s. = not significant).

To characterize ARHGAP18 localization in wild-type epithelial cells, we used immunofluorescent staining of WT mouse intestinal tissue against ARHGAP18. We co-stained with phalloidin (actin), DAPI (nucleus), and Ezrin (microvilli) to characterize which domains ARHGAP18 was present in epithelial cells of the gut. Super Resolution by Optical Pixel Reassignment (SORA) confocal images revealed that ARHGAP18 is enriched but not exclusively localized to the apical membrane of wild type (WT) epithelial cells (Figure 1, A–C). The enrichment of ARHGAP18 at the apical surface corresponds with our group's recent characterization of the regulation of microvilli through direct binding of ARHGAP18 to Ezrin (Lombardo *et al.* 2024). Quantification of the super-resolved ARHGAP18 localization showed that while ezrin and actin staining were strongly depleted from the nucleus, ARHGAP18 maintained a moderate signal in the cytoplasm and a lower but present signal in the nucleus (Figure 1B).

### Overexpression and knockout of ARHGAP18 both induce increased myosin light chain phosphorylation

We sought to understand ARHGAP18’s potential role in regulating RhoA in the cytoplasm and nucleus through Western blotting of whole cell lysates. Previously, we produced and validated CRISPR/CAS9 knock out of ARHGAP18 in human epithelial Jeg-3 cells (Figure 1D) and characterized its apical and microvilli-specific function (Lombardo *et al.*, 2024). In microvilli, ARHGAP18 acts to suppress active RhoA, leading to reduced ROCK activation and lower myosin light chain phosphorylation (pMLC). We hypothesized that the overexpression of ARHGAP18 would result in reduced pMLC by decreasing the active fraction of RhoA at the apical membrane through ARHGAP18’s GAP activity. Counterintuitively, we found that cells overexpressing ARHGAP18 and cells lacking ARHGAP18 both show increased phosphorylation of myosin light chain (Figure 1, E and F). While surprising to us initially, we found that these results were supported by several previous characterizations in various model species of ARHGAP18’s unexpected phenotypes in response to knockdown or overexpression (Neisch *et al.*, 2013; Porazinski *et al.*, 2015; Lay *et al.*, 2019). While pMLC was sensitive to the abundance or absence of ARHGAP18, total expression levels of NM-Myo-2B, total MLC, and actin are unchanged across these conditions (Figure 1E). The increase in pMLC in ARHGAP18 overexpressing cells indicates a potential mechanism in which pMLC could be regulated independently of ARHGAP18’s GAP function.

### Cells lacking ARHGAP18 have disorganized basal actin and a loss of focal adhesions

**FIGURE 2:**
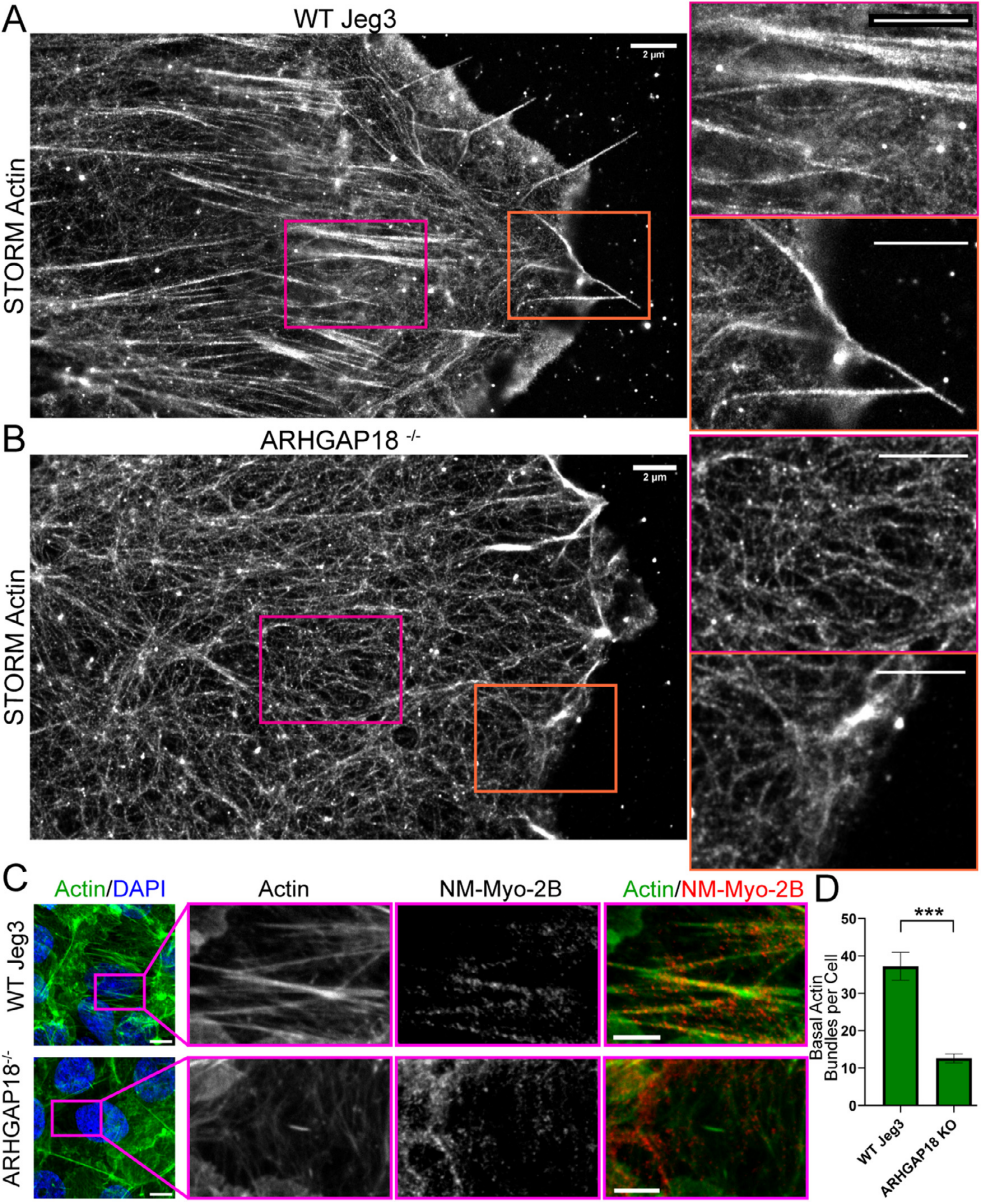
STORM Reconstruction of Individual Actin Architecture in WT and ARHGAP18 KO Jeg-3 Cells. A) STORM reconstructions of phalloidin stained actin in fixed Jeg-3 WT cells. Scale bar 2 µm; inset 2 µm. B) STORM reconstructions of phalloidin stained actin in fixed ARHGAP18 KO Jeg-3 cells. Scale bar 2 µm; inset 2 µm. C) Immunofluorescence imaging of actin and Non-Muscle Myosin-2B (NM-Myo-2B) in WT and ARHGAP18 KO cells. Scale bar 10 µm; inset 5 µm. D) Quantification of NM-Myo-2 actin colocalized bundles in WT and ARHGAP18 KO cells. Bars indicate Mean ± SEM; Significance by t-test (*n* = 32 cells, *** = *p* ≤ 0.001).

Increased pMLC in the absence of ARHGAP18 is expected to result in increased stress fiber formation through the activation of NM-Myo-2. However, multiple independent investigations, including our own, had identified the loss of stress fibers in cells with reduced or lacking ARHGAP18 (Neisch *et al.*, 2013; Porazinski *et al.*, 2015; Lay *et al.*, 2019; Lombardo *et al.*, 2024). The true structure of actin filaments is well below the diffraction-limited resolution of light, which forms dense arrays of filaments at the plasma membrane cortex interface. To characterize the actin filament organization at the basal surface of cells lacking ARHGAP18, we employed Stochastic Optical Reconstruction Microscopy (STORM) to resolve the individual actin architecture to a spatial X-Y resolution of less than 40 nm. Jeg-3 cells were plated at a sub-confluent density and imaged at the leading sections of cell groupings where the cells were expanding into open areas in the culture dishes. Under these conditions, Jeg-3 WT cells showed numerous stress fiber bundles at the basal surface of the cell (Figure 2A). Actin filaments transitioned to lateral filaments running parallel to the plasma membrane and eventually to a canonical leading edge comprised of both cortical branched individual filaments and filopodia at the cell's plasma membrane. The orientation of the stress fibers and filopodia generally aligned with the axis going from the cell center to the cell exterior. In contrast to the WT actin organization, cells lacking ARHGAP18 exhibit a striking absence of both stress fibers and filopodia (Figure 2B). The actin of ARHGAP18 KO cells was organized into small bundles and long individual filaments. Notably, the orientation of the ARHGAP18 KO cells’ actin was substantially less aligned in the general axis going from the cell center to the cell exterior, as seen in the WT cells.

To further characterize the basal actin phenotype in cells devoid of ARHGAP18, we used immunofluorescent staining of NM-Myo-2B using spinning disk confocal microscopy. When levels of pMLC and active NM-Myo-2B are increased, the expectation would be an increase in the straight-puncta localization of NM-Myo-2B to contractile actin bundles associated with focal adhesions (Beach *et al.*, 2017). Stress fibers from WT cells showed actin colocalized with repeating striations of NM-Myo-2B, indicative of active, contractile fibers (Figure 2C). NM-Myo-2 still colocalized to actin in ARHGAP18-deficient cells but organized into smaller, more diffuse puncta (Figure 2, C and D). We previously reported that ARHGAP18-deficient cells were nearly twice as stiff as WT cells when measured by force indentation using atomic force microscopy (Lombardo *et al.*, 2024). Given these data, combined with our western blotting of pMLC, we concluded that NM-Myo-2B was still active in cells lacking ARHGAP18 and capable of forming into contractile bundles, but that the force-producing activity was not organizing the actin into large stress fiber bundles seen in the WT cells through an unknown mechanism.

### Cells lacking ARHGAP18 have transcriptional changes in actin and proliferation regulators

**FIGURE 3:**
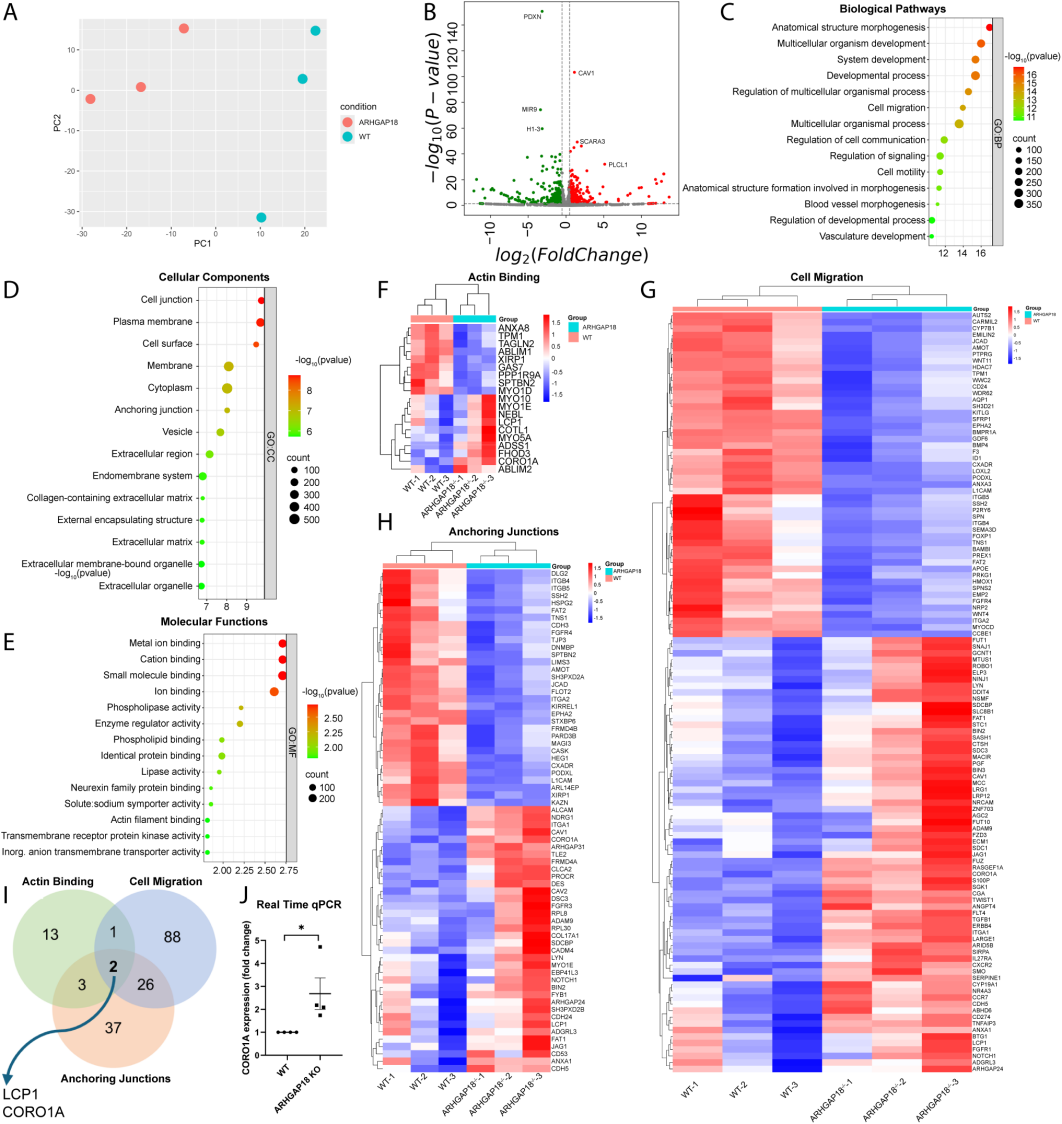
ARHGAP18 total RNA sequencing shows changes in transcription of actin regulatory processes. A) Principle Component Analysis (PCA) from total RNA sequencing results obtained from WT Jeg-3 and ARHGAP18 KO cells (*n* = 3). B) plot of adjusted p-value vs. log fold change in expression (volcano plot) with select genes identified. C) Go terminology analysis of biological pathways. Color indicates *p*-value, representing the likelihood that a pathway is altered in ARHGAP18 KO versus WT Jeg-3 cells while bubble size indicates the number of genes identified with altered expression. D) GO terminology analysis of Cellular components. E) GO terminology analysis of Molecular Functions. F) Heat map of the genes where the gene product is a known or predicted actin binding protein in each of the three biological replicates from WT Jeg-3 or ARHGAP18 KO cells. Color indicates fold change where blue is lower expression and red is increased expression compared with the other group. G) Heatmap similar to (F) for Cell Migration gene products H) Heatmap similar to (F) for Anchoring Junctional gene products. I) Venn diagram identifying genes that are combinations of the terms shown in (F–H). LCP1 and CORO1A's gene products bind actin and have been characterized to play a role at anchoring junctions and in cell migration. J) Real Time qPCR analysis of CORO1A expression (fold change) between Jeg-3 WT and ARHGAP18 KO cells. Conditions are significant by ratio paired *t* test, *n* = average of 4 replicates (* = *p* ≤ 0.05).

We hypothesized that the loss of basal actomyosin bundles observed at the basal surface of cells devoid of ARHGAP18 may be a result of changes in regulation at the transcriptional level. The molecular pathways driving the basal actomyosin defect were unknown, so we sought to perform a screen for transcriptional pathways that were altered in the cells lacking ARHGAP18. To characterize changes at the transcriptional level, we performed total RNA sequencing (RNAseq) on WT-Jeg-3 and ARHGAP18 KO cells. We confirmed expression changes between WT and ARHGAP18 KO cells using PCA analysis (Figure 3A). The analysis revealed two distinct clusters corresponding to WT Jeg-3 and ARHGAP18 KO samples, suggesting clear transcriptomic differences between the two groups. A total of 889 differentially expressed genes, with 551 upregulated and 368 downregulated genes, were identified in ARHGAP18 KO cells compared with WT Jeg-3 cells. The distribution of these DEGs was displayed in a volcano plot (Figure 3B).

To further explore the impact of ARHGAP18 KO on overall cell functions, we performed Gene Ontology (GO) enrichment analysis and identified that across different GO categories, cell morphology, migration, junctional anchoring, and actin filament binding terms were significantly altered in cells lacking ARHGAP18 (Figure 3, C–E). Additionally, numerous terms related to microvilli and the apical plasma membrane, including ion binding, lipid binding, and plasma membrane binding, were also enriched. To investigate the expression changes of specific genes associated with these terms across all of our samples, we plotted the expressional patterns of each gene into clustered heatmaps (Figure 3, F–H). Microvilli-specific regulators included multiple myosin motor proteins responsible for membrane-to-plasma membrane linkages (Myo1E and Myo1D) and transport of lipid and protein vesicles inside microvilli (Myo10). By comparing the proteins that meet the shared characteristics of anchoring junctions, cell migration, and actin binding (Figure 3I) we identified LCP1, also known as L-plastin, as upregulated in ARHGAP18-deficient cells. Plastin-1 is a predominant actin bundler in microvilli (also called Fimbrin), while L-plastin is associated with tumorigenesis when expressed outside of bone. A second gene, CORO1A (Coronin 1), was also identified, which is involved with RhoA-dependent signaling (Priya *et al.*, 2016). We validated the RNA-seq result for CORO1A by RT-qPCR and found that the expression changes between WT and ARHGAP18 KO cells were statistically significant (Figure 3J). These results are in agreement with our previous characterization of ARHGAP18 in the biogenesis and maintenance of microvilli at the apical membrane through RhoA (Lombardo *et al.*, 2024).

Further analysis of the individual gene products associated with actin binding, cell migration, and anchoring junctions identified proteins associated with actin and cell adhesion at the basal surface. Most notable among these were the alterations in the expression of Integrins (ITGA1, ITGA2, ITGB5, and ITGB4) and other focal adhesion gene products such as TNS1 (Figure 3, F–H). To further investigate the relationship between genes with altered expression in ARHGAP18 KO, we performed network analysis using Ingenuity Pathway Analysis (IPA). Surprisingly, network analysis revealed that most expression changes found in our RNAseq screen did not occur through known protein-protein interactions or directly through RhoA signaling (Supplemental Figure S1). These results promoted the hypothesis that the expression changes in ARHGAP18 KO cells derive from alterations in transcription rather than through direct protein–protein interactions.

To identify which transcription factor pathways could be altered in cells lacking ARHGAP18, we manually characterized the known signaling pathways involved with all down-expressed genes related to cell migration (Supplemental Table S1). We found that of these genes, more than half of them were associated with WNT, HIPPO, or MAPK/PI3K signaling pathways, and multiple were direct YAP/TAZ effectors (Supplemental Table S1). From this analysis, we formed the hypothesis that loss of ARHGAP18 influences HIPPO-dependent transcriptional signaling. Additionally, we hypothesized that focal adhesions would be severely disrupted at the basal membrane. Our RNAseq data alone could not independently confirm if the alterations to transcriptional signaling and expression of actin cytoskeleton proteins were through a Rho-dependent or Rho-independent mechanism. Instead, these data provided an informed hypothesis as to which signaling pathways are altered in the ARHGAP18 KO cells as compared with WT Jeg-3 cells.

**FIGURE 4:**
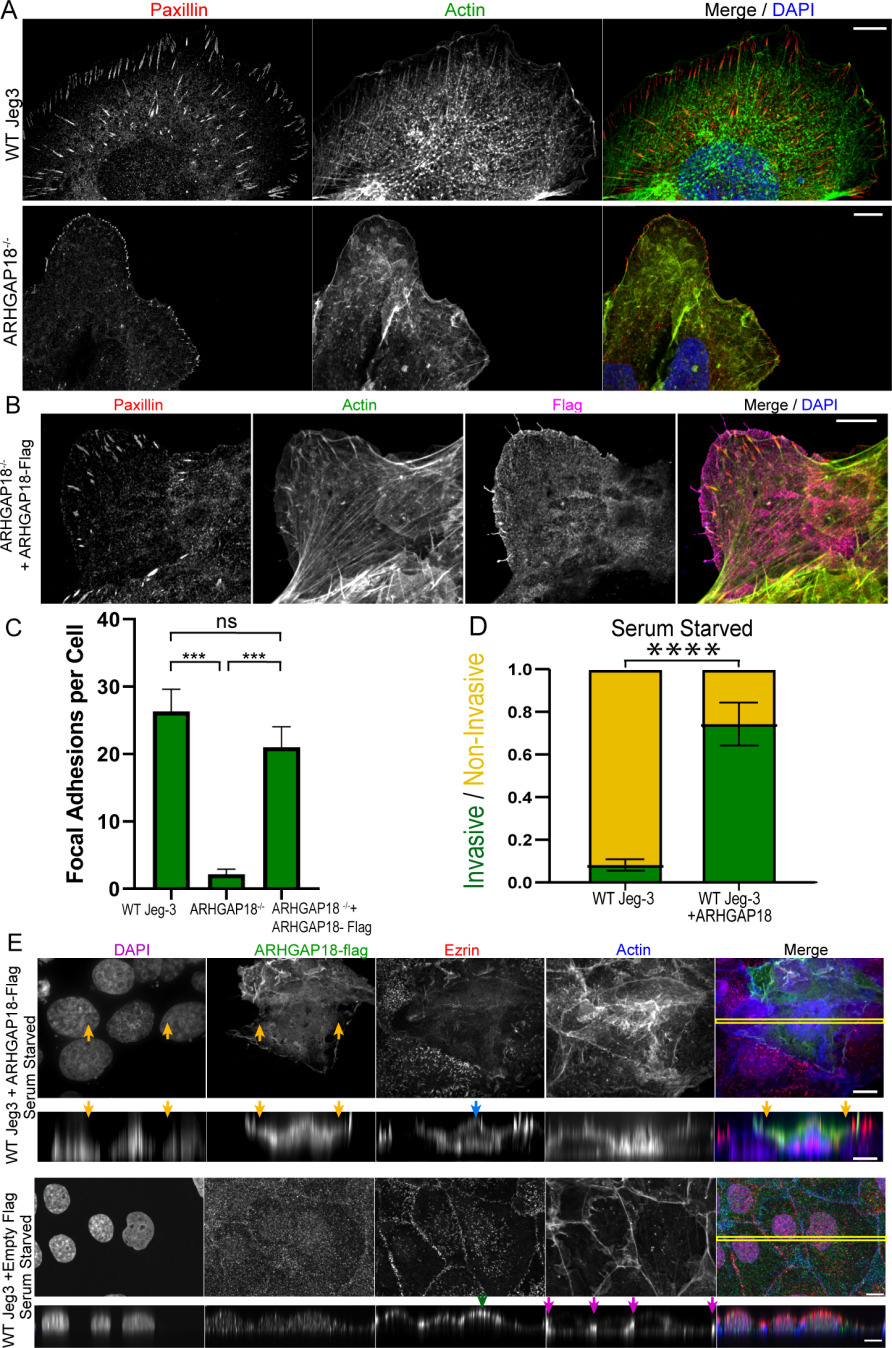
Loss of ARHGAP18 disrupts NM-Myo-2B and paxillin associated actin structures at the basal surface. A) Spinning disk confocal SORA immunofluorescent imaging of paxillin and actin in Jeg-3 WT and ARHGAP18 KO cells. ARHGAP18 KO shows ablation of most paxillin focal adhesions. Scale bar 10 µm. B) Spinning disk confocal SORA immunofluorescent imaging of ARHGAP18-flag, anti-paxillin, and actin in ARHGAP18 KO + ARHGAP18-Flag cells (rescue). Scale bar 10 µm. C) Quantification of the number of paxillin-stained focal adhesions per cell. Bars are Mean ± SEM. conditions are significant by unpaired *t* test; WT *n* = average of four replicates, ARHGAP18^-/-^ *n* = average of seven replicates, ARHGAP18^-/-^ +ARHGAP18-Flag *n* = average of seven replicates (** = *p* ≤ 0.01, *** = *p* ≤ 0.001, n.s. = not significant). D) Percent invasive/ noninvasive cells quantification of serum starved WT Jeg-3+ empty vector flag and WT Jeg-3 +ARHGAP18 flag cells. Bars are Mean ± SEM. Conditions are significant by unpaired *t* test (*p* ≤<0.0001; *n* = 144 cells over three biological replicates). E) Spinning disk confocal immunofluorescent imaging of DAPI, ARHGAP18-flag, anti-ezrin, and actin in serum starved Jeg 3 WT+ ARHGAP18-Flag (overexpression) and WT Jeg-3 + Empty Vector flag. The lower image rows show an X-Z dimension confocal slice of the same image, highlighting the invasion of overexpressing cells into neighboring cells or the lack of invasion in empty vector cells. Yellow arrows indicate the sections of the overexpressing cell invading over neighboring cells’ nucleus. Blue arrow indicates two stacked apical membranes by ezrin staining with the lower membrane from WT cells underneath the upper membrane from an ARHGAP18 overexpressing cell. The green arrow indicates the absence of stacked apical membranes in the ezrin-stained section, and the pink arrows show well-defined junctional actin staining from WT Jeg-3 empty flag cells. Scale bars 10 µm.

Following this analysis, we tested the hypothesis that focal adhesions would be disrupted at the basal membrane. However, staining transmembrane integrins to visualize changes predicted by our RNAseq analysis presented significant technical challenges. Specifically, the structural localization of immuno-antigen sites within transmembrane or extracellular domains required fixation conditions that disrupted actin filament organization, preventing us from performing colocalization experiments. We therefore used Paxillin staining as a general marker for focal adhesions to test the prediction from our RNAseq screen that focal adhesion would be disrupted in cells lacking ARHGAP18. immunofluorescence SORA imaging of Paxillin in WT cells showed long focal adhesions tethered to stress fibers at the basal surface (Figure 4A). Imaging of ARHGAP18 KO cells showed small Paxillin puncta localized to the edge of cells, with nearly no observed Paxillin colocalized with stained actin bundles (Figure 4A). Expression of full-length human ARHGAP18-flag in ARHGAP18 KO cells rescued both basal actin bundles and paxillin focal adhesions (Figure 4, B and C). Using our super-resolved SORA images, we defined colocalized actin bundles with paxillin patches as focal adhesions and quantified the number of focal adhesions per cell. This quantification revealed a significant decrease in focal adhesions in ARHGAP18 KO cells compared with WT cells (Figure 4C).

### ARHGAP18 overexpressing cells show an invasive phenotype

We sought to characterize the defects of pMLC overactivation, loss of basal actomyosin bundles, and paxillin mislocalization in cells overexpressing ARHGAP18-flag. However, we discovered that WT cells overexpressing ARHGAP18 would often break from the surrounding Jeg-3 cells’ monolayer to form three-dimensional stacks of cells (Figure 4, D and E). This characteristic was not typically seen in monolayered WT and ARHGAP18 KO cells (Figure 2C). Due to the 3D stacking of the overexpression cells, we considered these cells to be potentially invasive as defined by their ability to move beyond their typical single monolayer boundary. Specifically, we defined the invasive phenotype as cells in a monolayer that projected their cytoplasm greater than 2 µm on top of or below neighboring cells as detected by actin, DAPI, ezrin, and flag fixed cell staining (Figure 4, D and E). We were unable to precisely quantify paxillin-stained focal adhesions in this condition, as we lacked confidence in our ability to accurately identify the fluorescent signal of the paxillin patches from stacked cells on top of each other. To quantify the phenotype, we counted the number of cells in a monolayer that projected their cytoplasm greater than 2 µm on top of or below neighboring cells. We used transient transfection of ARHGAP18-flag into WT cells, which allowed us to compare the behavior of WT cells side-by-side with ARHGAP18 overexpressing cells (Figure 4, D and E). Spinning disk confocal Z-dimensional slicing of the actin on these cells indicated that large basal actin bundles were present in the ARHGAP18 overexpression cells (Figure 4E). We observed that cells overexpressing ARHGAP18 invade their neighboring cells’ monolayer, where we observed overexpressing cells invading the adjacent WT cells (Figure 4E, yellow arrows). By this measure, 74% ± 10.0% of ARHGAP18 overexpressing cells displayed an invasive phenotype. In these invasive cells, Z-dimensional confocal slices of ARHGAP18-overexpressing cells showed two ezrin apical plasma membrane layers stacked vertically, indicating that the cells are organizing into three-dimensional stacks (Figure 4E, blue arrow). In contrast, only 8% ± 2.6% of the WT empty flag controls invaded neighboring cells. Instead, the remaining 92% of WT cells maintained well-defined actin belts at points of cell–cell contact (Figure 4E, pink arrows) and the presence of a single band of apically stained ezrin (Figure 4E, green arrows), indicating a well-ordered monolayer. These data support the conclusion that ARHGAP18 acts to regulate basal and junctional actin.

### ARHGAP18 forms a complex with Merlin

**FIGURE 5:**
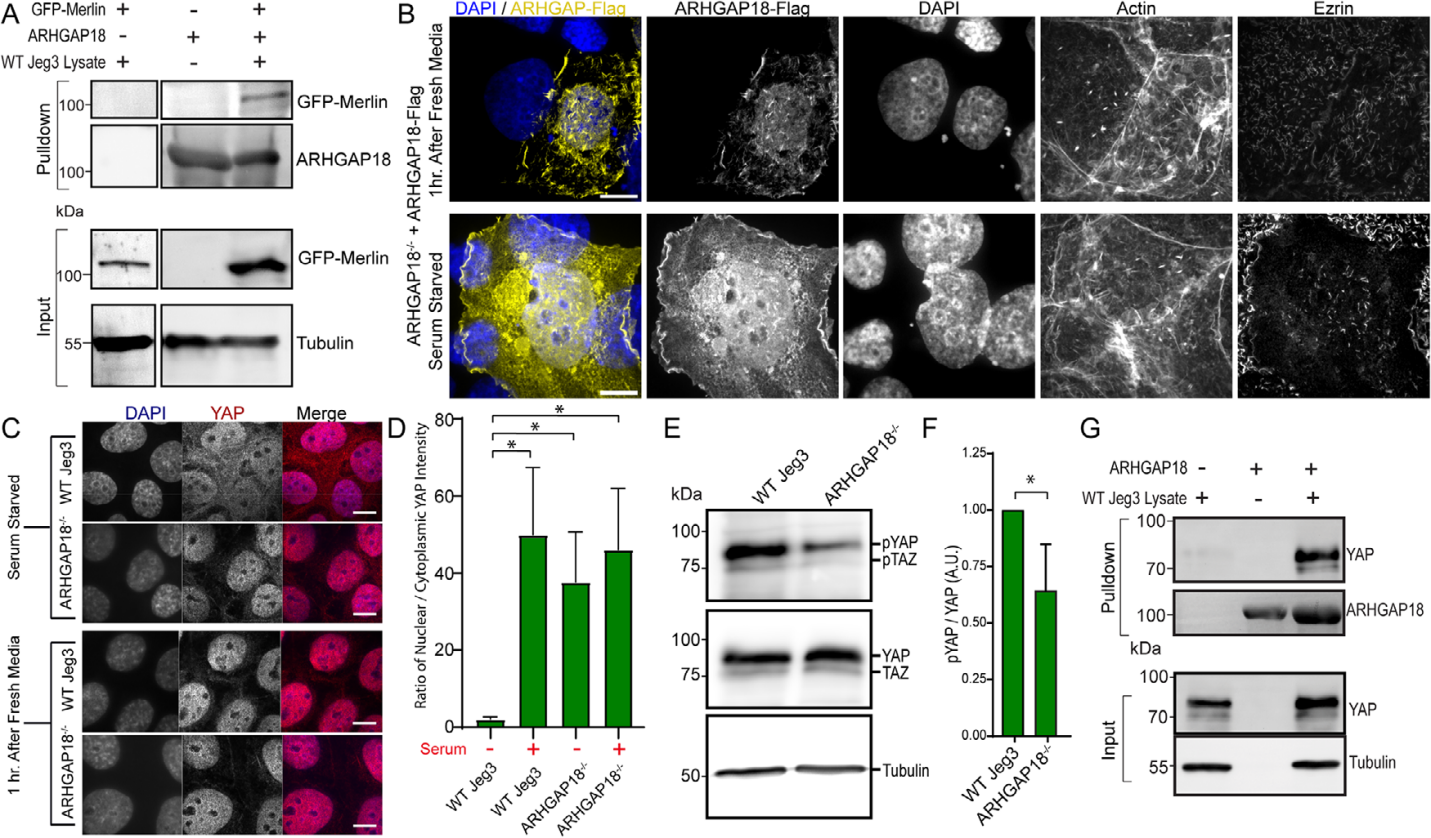
ARHGAP18 complexes with merlin and YAP affecting its localization throughout the cell based on nutrient availability. A) Western blot of an ARHGAP18 pulldown from WT Jeg-3 cells with or without expression of GFP-merlin. B) immunofluorescence imaging of ARHGAP18-flag rescue of ARHGAP18 KO cells in the serum starved or 1 h after reintroduction of serum. ARHGAP18’s localization includes the cytoplasm in the serum starved state. Ezrin staining of microvilli shows lower levels of microvilli in both conditions. Scale bar 10 µm. C) Immunofluorescence imaging of YAP in WT and ARHGAP18 KO cells in the serum starved state or 1 h after reintroduction of serum. YAP localization to the cytoplasm during serum starvation is lost in ARHGAP18 KO cells. Scale bar 10 µm. D) Quantification of ratio of nuclear to cytoplasmic YAP intensity from Figure (C). Bars are Mean ± SEM. Conditions are significant by unpaired *t*-test (*p* ≤ 0.05) *n* = 3 E) Western blot of lysate from WT or ARHGAP18 KO cells shows a decrease in the phosphorylation of YAP and TAZ (pYAP/ pTAZ) in the knockout cells. F) Quantification of the band intensity of pYAP/YAP as shown by the blot in (E). Bars indicate Mean ± SEM; conditions are significant by paired *t*-test (*p* ≤ 0.05) *n* = 37 cells. G) Pulldown experiment using an ARHGAP18-agarose column. YAP does not bind to the column agarose alone nor is YAP present on the purified ARHGAP18 column before the addition of WT Jeg-3 lysate. When Jeg-3 lysate passed over the ARHGAP18 column endogenous YAP stably binds.

A second hypothesis arising from our RNAseq screen was the hypothesis that loss of ARHGAP18 influences HIPPO-dependent transcriptional signaling (Figure 3; Supplemental Table S1). Multiple prior studies also indicated that ARHGAP18 interacts through the Hippo pathway to regulate transcription of actin organization and cell proliferation (Porazinski *et al.*, 2015; Coleman *et al.*, 2020). We'd earlier identified that ARHGAP18 bound to the FERM domain of ERMs (A14 to I20 of human Ezrin) through a N-terminal extension distinct from the RhoA GAP domain (V10 to S17 of human ARHGAP18; Lombardo *et al.*, 2024). We hypothesized that ARHGAP18 could bind Merlin as its FERM domain closely resembles Ezrin. Sequence alignment identified a motif (T28-F35 in Human Merlin) conserved across human Merlin, Ezrin, Radixin, and Moesin, overlapping the known ARHGAP18 binding site identified earlier. Merlin, which localizes to cell–cell junctions, acts upstream of YAP/TAZ through the HIPPO pathway. We tested if ARHGAP18 could form a complex with Merlin using a pull-down approach by passing lysate from WT Jeg-3 cells expressing a tagged Merlin construct over an immobilized ARHGAP18 column. We found that Merlin expressed in human Jeg-3 cells bound to the ARHGAP18 column (Figure 5A).

To validate this interaction in cells, we sought to identify whether ARHGAP18 was localized to junctions where Merlin is active. Merlin is activated and localized to junctions upon signaling, promoting growth and proliferation; among these signals is the availability of growth factors and other components of serum (Bretscher *et al.*, 2002). We hypothesized that since ARHGAP18 formed a complex with Merlin, ARHGAP18’s localization may be localized to junctions under conditions that promote Merlin activation. We used the rescue of ARHGAP18 KO cells with a flag-tagged ARHGAP18 to image the localization of ARHGAP18 in serum-starved versus cells supplemented with fetal bovine serum (FBS) for 1 h following serum starvation (Figure 5B). Maximum projection images in both conditions showed localization of ARHGAP18 to microvilli at the apical surface, along with the rescue of increased microvilli formation in ARHGAP18 KO cells as we've previously reported (Figure 5B; Lombardo *et al.*, 2024). However, in cells supplemented with serum, ARHGAP18 localized strongly to the nucleus and junctions while being almost entirely excluded from the cytoplasm. When cells were deprived of serum, ARHGAP18’s localization shifted to include substantial cytoplasmic staining (Figure 5B). Interestingly, ARHGAP18’s localization to microvilli and its reduction of the formation of microvilli were maintained in both conditions, indicating that the microvilli-specific functions were independent of serum availability. Together, these data indicate that ARHGAP18 localization to junctions is modulated by serum conditions. While Merlin localization was not directly assessed in these experiments, the observed redistribution is consistent with a potential role for ARHGAP18 in junction-associated signaling. In addition, the nuclear and cytoplasmic shifts suggest ARHGAP18 may have context-dependent regulatory functions.

### ARHGAP18 regulates YAP phosphorylation and nuclear localization

We hypothesized that ARHGAP18’s localization to the nucleus may involve interaction with YAP as had previously been proposed (Porazinski *et al.*, 2015; Coleman *et al.*, 2020). In serum-starved WT cells, YAP is phosphorylated and localized to the cytoplasm. Upon reintroduction of serum, YAP phosphorylation is reduced, and it is shuttled to the nucleus, where it acts to promote cell proliferation and actin polymerization. We tested whether YAP shuttling between the nucleus and cytoplasm in response to serum availability was altered in Jeg-3 cells lacking ARHGAP18. We used immunofluorescent staining of YAP and TAZ in cells fixed in either serum-fed or serum-starved states. In WT serum-starved cells, YAP/TAZ showed diffuse staining throughout the cytoplasm and nucleus (Figure 5, C and D). Upon reintroduction of serum into the media, YAP/TAZ's cytoplasmic localization was lost within one hour, and only nuclear staining remained. In ARHGAP18 KO cells, YAP/TAZ remained predominantly in the nucleus in both the serum-starved and serum-present media states. Using lysates from the serum-starved cells, we tested by western blotting the phosphorylation state of YAP and TAZ (Figure 5E). While the total expression of YAP and TAZ remained the same in WT and ARHGAP18 KO cells, the phosphorylation of YAP and TAZ was significantly reduced in the cells lacking ARHGAP18 compared with WT levels (Figure 5, E and F).

We hypothesized that ARHGAP18 may also form a complex with YAP that influences YAP's cytoplasmic localization. To test this hypothesis, we passed WT Jeg-3 lysates over the immobilized ARHGAP18 column to probe for binding of native proteins to ARHGAP18. We detected that endogenous YAP from WT Jeg-3 cells bound to immobilized ARHGAP18 (Figure 5G). We concluded that YAP's localization to the cytoplasm in the serum-starved state depended on the formation of a complex including both YAP and ARHGAP18. The combined results involving ARHGAP18’s interactions with YAP and Merlin indicate that ARHGAP18 acts as part of a Hippo signaling component involved in the cytoplasmic localization of YAP to control polarized actin cytoskeletal structures.

### Dependence of apical and basal actin structures on ARHGAP18 GAP-dependent signaling

**FIGURE 6:**
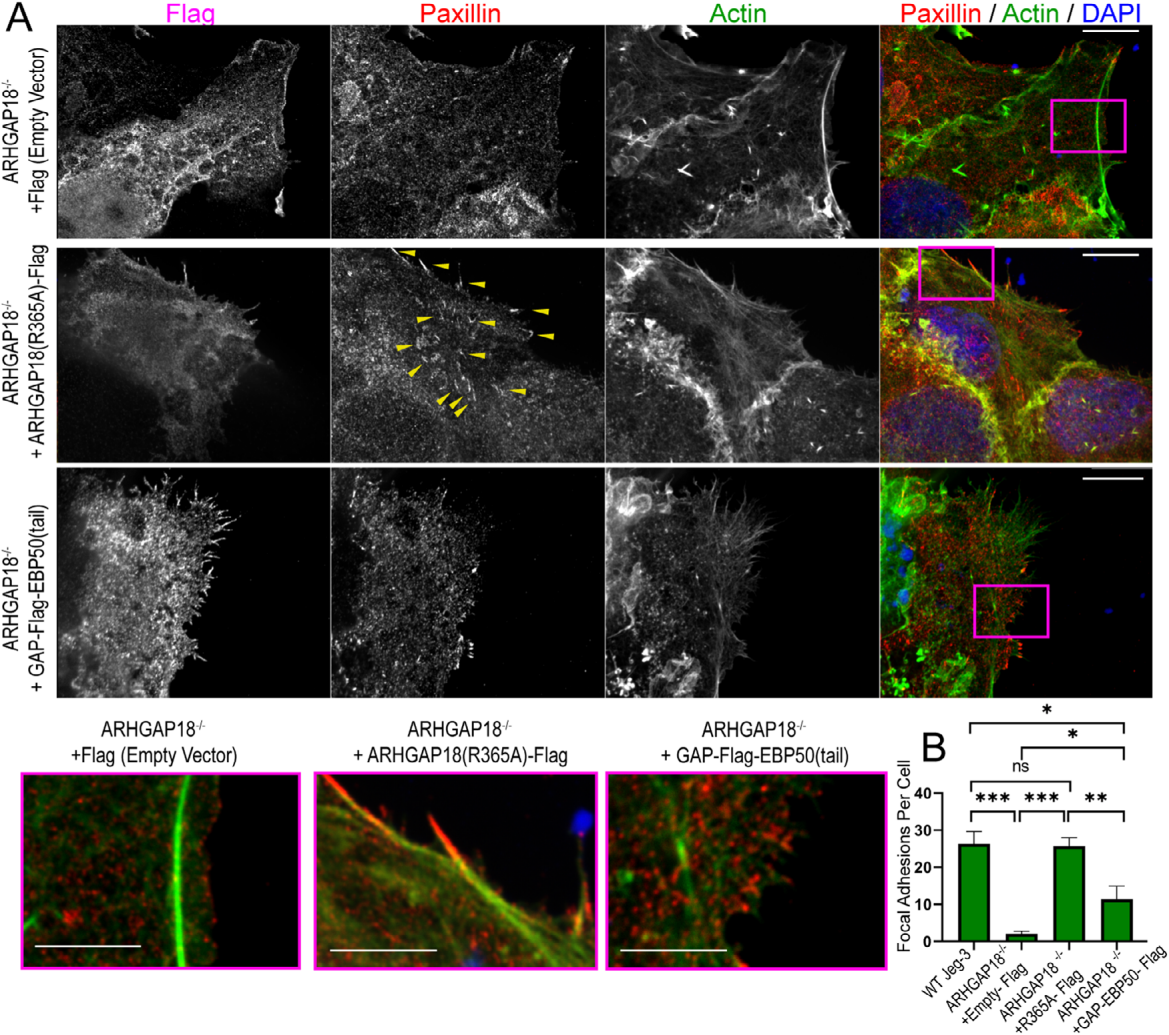
ARHGAP18 KO cell's focal adhesion phenotype is rescued by a Rho GAP deficient ARHGAP18 mutant. A) Spinning disk confocal SORA immunofluorescent imaging of flag, anti-paxillin, and actin in ARHGAP18 KO + Flag (empty vector), ARHGAP18 KO+ ARHGAP18(R365A)-Flag, and ARHGAP18+ GAP-Flag- EBP50(tail). Yellow arrows indicate paxillin stained focal adhesions, which are rescued in the ARHGAP18 KO+ARHGAP18(R365A)-Flag condition. Scale bar 10 µm; inset 5 µm. B) Quantification of paxillin stained focal adhesions per cell from (A) and WT Jeg-3 duplicated from Figure 4C for comparison. Bars are Mean ± SEM. Conditions are significant by unpaired *t* test, WT Jeg-3 *n* = Average of four biological replicates, ARHGAP18 KO conditions *n* = average of seven biological replicates, (* = *p* ≤ 0.05, ** = *p* ≤ 0.01, *** = *p* ≤ 0.001, n.s. = not significant).

It was unclear if all the cytoskeletal changes we characterized in cells lacking ARHGAP18 were dependent on its RhoA GAP activity. One alternative was that the binding of ARHGAP18 to the HIPPO components conferred a Rho GAP independent effect. To test whether ARHGAP18 had the potential to regulate the actin cytoskeleton through a Rho GAP-independent mechanism, we introduced a point mutation in the conserved catalytic arginine (position 365) within the RhoA GAP domain, substituting it with an alanine (R365A). This mutation is known to ablate ARHGAP18’s GAP activity (Barrett *et al.*, 1997; Maeda *et al.*, 2011). Expression of human ARHGAP18(R365A)-Flag in ARHGAP18 KO cells resulted in a restoration of paxillin-stained focal adhesions and organized basal actin bundles to WT levels (Figure 6A, yellow arrows; Figure 6B). Expression of an empty flag vector in ARHGAP18 KO cells did not restore basal actin or focal adhesions (Figure 6, A and B). These results indicated that ARHGAP18 at least partially regulated basal actin-based structures through a mechanism independent from its RhoA GAP activity.

We hypothesized that the Rho GAP-independent activity of ARHGAP18 involved its N-terminal region; ablation of this region results in a diffuse localization of the RhoA GAP domain and inactivation of RhoA activity throughout the entire cell (Lombardo *et al.*, 2024). We aimed to separate ARHGAP18’s RhoA GAP activity at sites of active ERMs from its potential ability to bind additional actin-regulating proteins. To test if apical RhoA signaling alone could rescue the basal actin defect in ARHGAP18-deficient cells, we expressed a synthetic GAP that targeted to ERMs in ARHGAP18 KO cells. We generated a construct that fused the GAP domain of ARHGAP18 to a 37 amino acid ezrin binding motif from Ezrin Binding Phosphoprotein of 50kDa (EBP50) through a flexible linker (GAP-Flag-EBP50(tail)) (Lombardo *et al.*, 2024). Expression of GAP-Flag-EBP50(tail) in ARHGAP18 KO cells showed disorganized basal actin like the ARHGAP18 KO cells (Figure 6A). However, it resulted in a partial rescue of focal adhesions but did not restore the number of focal adhesions to WT levels (Figure 6, A and B). We concluded that appropriate apical RhoA GAP regulation by ARHGAP18 is necessary for the full establishment of apical actin organization. Disruption of apical RhoA regulation can have effects distal to the location, and these data suggest that basal actin organization and focal adhesion formation can be influenced by apical RhoA regulation. However, the GAP-Flag-EBP50(tail) rescue was not sufficient to fully rescue the number of paxillin-stained focal adhesions and basal actin bundles in ARHGAP18 KO cells (Figure 6, A and B). Thus, we concluded that ARHGAP18 additionally possessed the ability to partially regulate basal actin organization and focal adhesions through a RhoA GAP-independent mechanism. Further, this RhoA GAP-independent mechanism depended on domains of ARHGAP18 outside of the GAP domain, and its ERM-FERM localizing motif.

## DISCUSSION

Humans have 20 Rho/Ras family of GTPase genes, where nucleotide state transitions are accelerated by a plethora of guanine nucleotide exchange factors (GEFs) and GTPase-activating proteins (GAPs). This genetic complexity, combined with partial overlapping molecular functions, has presented significant challenges to the characterization of the molecular functions of individual GAPs and GEFs in humans. For example, of the approximately 83 GEFs in humans, at least 24 have specific or partial activity for a single GTPase, RhoA (Vigil *et al.*, 2010). Humans also have genes for three dissociation inhibitors (RhoGDIs), which regulate Rho family localization through membrane binding. Thus, much of our foundational understanding derives from careful experimentation in more genetically advantageous model systems (Etienne-Manneville and Hall, 2002). Here, we use CRISPR-mediated knockout of ARHGAP18 in human epithelial cells to examine its role in actin cytoskeletal organization. We find that ARHGAP18 associates with the Hippo pathway components YAP and Merlin, suggesting potential points of crosstalk between these signaling pathways. These associations correlate with changes in actin organization and cell morphology, consistent with ARHGAP18 contributing to multiple aspects of cytoskeletal regulation. While these findings support a model in which Rho family regulators participate in complex and multifaceted cellular functions, the precise mechanisms linking these pathways remain to be fully defined.

**FIGURE 7:**
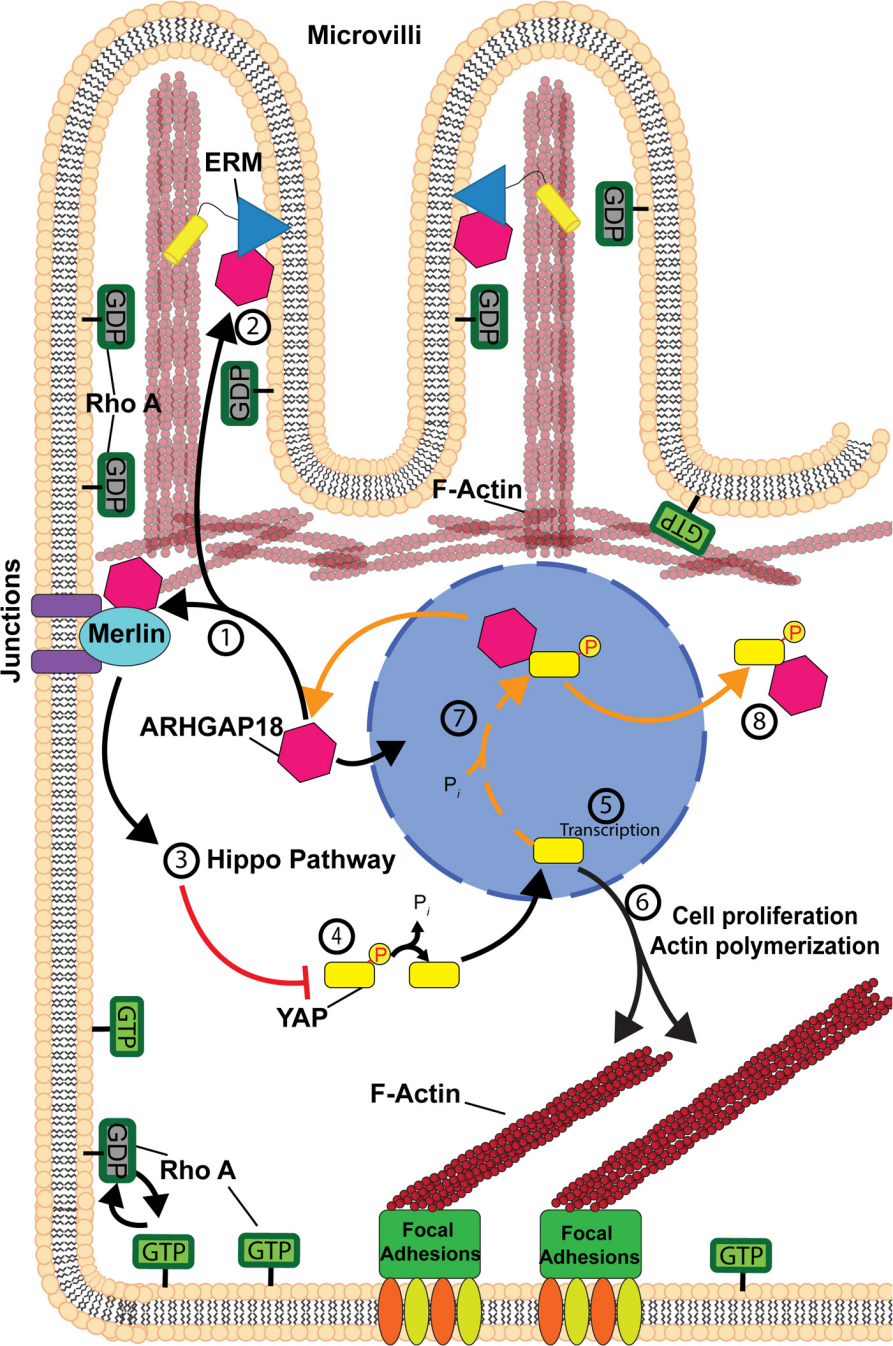
Theoretical working model of ARHGAP18’s interactions with RhoA and Hippo signaling pathways in human epithelial cells. Under normal cellular conditions (black arrows), ARHGAP18 localizes to the nucleus, cell junctions, and microvilli. ARHGAP18 forms complexes with (1) Merlin at cell junctions and (2) ERMs in microvilli through direct binding to their conserved FERM domains. Once recruited to junctions by Merlin, ARHGAP18 regulates RhoA locally. Merlin simultaneously coordinates mechanosensation and proliferation signaling through activation of the Hippo pathway. (4) Hippo signaling regulates the phosphorylation of YAP, where the active, dephosphorylated state is shuttled to the nucleus. (5) Active YAP/TAZ coordinates transcriptional changes, which result in (6) expression of proteins regulating the actin cytoskeleton and proliferation. 7) Alternatively, when Hippo signaling suppresses YAP activation (orange arrows), YAP is phosphorylated through an ARHGAP18 dependent mechanism. 8) ARHGAP18 complexes with YAP, which may be required for proper phosphorylation and eventual shuttling of YAP to the cytosol for degradation. Loss of ARHGAP18 is correlated with persistent localization of YAP to the nucleus with constitutively active proliferation signaling in addition to misregulation of RhoA at junctional and apical membranes.

ARHGAP18 KO cells exhibit a near total loss of stress fibers (Figure 2) despite increased cellular levels of pMLC (Figure 1, E and F; Lombardo *et al.*, 2024). Additionally, focal adhesions and basal actin bundles are restored to WT levels when the ARHGAP18(R365A) GAP-ablated mutant is expressed in ARHGAP18 KO cells (Figure 6, A and B). These results represent the strongest argument that ARHGAP18 functions in additional pathways to RhoA/C alone. Our data suggest that at least one of the alternative pathways is through ARHGAP18’s interaction with YAP and Merlin. From these data, we conclude that ARHGAP18 has important functions in both RhoA signaling through both its GAP activity and in Hippo signaling through additional binding partners. From our data, we propose a working theoretical model for how these two functions could be achieved through ARHGAP18 (Figure 7). One major component of this model is ARHGAP18’s ERM-FERM-activated RhoA GAP activity. In this component, RhoA binds to and activates LOK and its homologue SLK (Zaman *et al.*, 2021), which then activates ERMs through phosphorylation at the conserved T567 (in Ezrin) site. This distinct Rho-dependent signaling pathway, termed ROLE signaling for RhoA-LOK-Ezrin, exists independently from ROCK signaling. ROLE signaling is controlled through a negative feedback loop coordinated by ARHGAP18 inside microvilli (Figure 7; Lombardo *et al.*, 2024).

Our data raises several questions about how RhoA and YAP could be coordinated, and some steps in our proposed working model (Figure 7) will require additional study to mechanistically define. First, what is the effect of ARHGAP18 interacting with both Merlin and YAP? Loss of ARHGAP18 results in increased active RhoA at junctions (Lombardo *et al.*, 2024), promoting apical actin reorganization. Thus, it's reasonable to conclude that one of Merlin's functions as a scaffolding protein is to sequester ARHGAP18 to junctions to regulate RhoA locally (Figure 7). When proliferative signaling is low, Merlin is inactivated and dissociates from the junctional cytoskeletal structures. Inactivation of Merlin would mask the predicted binding site for ARHGAP18 in the conserved FERM domain shared with ERMs (Bretscher *et al.*, 2002). ARHGAP18 would then be free to complex with YAP in the cytosol. The shuttling we characterize of ARHGAP18 localizing from junctions to the cytoplasm in response to serum availability (Figure 5, A and B) matches this proposed theoretical mechanism (Figure 7).

Second, why is nonmuscle myosin 2 not producing actin bundles despite being active in cells lacking ARHGAP18? The parameter space for defining this phenotype makes experimentation extremely challenging, as actin bundlers, nucleators, severing proteins, and polymerization effectors may all be affected by the alterations to YAP, Merlin, ERMs, and RhoA in ARHGAP18 KO cells. The list of available mechanisms that YAP alone may induce under perpetual nuclear localization (Figure 5C) is daunting (Pocaterra *et al.*, 2020). However, our RNAseq analysis narrows the list of potential mechanisms (Figure 3). The heat maps in our RNAseq data show substantial changes in actin binding, anchoring junctions, and cell migration in response to the loss of ARHGAP18. Of the many genes in these categories that show significant change, only two genes fit into all three groups: LCP1 and CORO1A. LCP1 is a fimbrin-related actin bundling protein. Thus, the actin bundle phenotype observed in ARHGAP18-deficient cells (Figure 2, A and B) may be a result of expression changes derived from YAP/TAZ's co-transcriptional activity. One potential explanation supported by our RNAseq analysis is the loss of the mechanical attachment point for the stress fiber bundles at the basal membrane. Integrin expression is severely disrupted in ARHGAP18-deficient cells (Figure 3), which would deprive a forming actomyosin bundle from mechanically attaching to the plasma membrane. Validation of the direct mechanism by which YAP/TAZ transcriptional changes drive basal actin changes in ARHGAP18 KO cells will require further investigation based on predictions from our RNAseq results.

Regardless of the genetic background, a major technical challenge for the study of Rho-dependent signaling pathway's regulation of cellular actin architecture is that 1) actin is the most abundant protein in the cytosol, 2) actin forms three-dimensional interwoven webs of densely packed networks, 3) filaments can turn over on the scale of 30–60 s, and 4) the single filament structure is 7 nm across (two orders of magnitude below the diffraction limit of visible light). We've overcome these hurdles using super-resolution STORM microscopy (Xu *et al.*, 2012). While multiple previous studies have observed the change in actin bundles of cells with altered ARHGAP18 levels (Neisch *et al.*, 2013; Porazinski *et al.*, 2015; Coleman *et al.*, 2020), our super-resolution characterization allows for the dissection of the exact actin structures lost. Reorganization toward branched or individual filaments would dramatically alter the transport of intracellular cargos by myosin and microtubule-based motors (Heaslip *et al.*, 2014; Lombardo *et al.*, 2017; Lombardo *et al.*, 2019; Bensel *et al.*, 2024). Our STORM data indicates that not all actin or bundles are lost, as it appears by our own traditional confocal staining (Compare Figure 4A with Figure 2B). Instead, significant quantities of single filaments are maintained in areas that appear devoid of actin under conventional light microscopy. When these findings are combined with our RNAseq data, the molecular pathways responsible for these changes are associated with a combination of RhoA signaling and pathways both upstream and downstream of YAP/TAZ. Our data indicate that the loss of ARHGAP18 switches the epithelial cell's basal actin signaling toward a state where the actin architecture resembles a leading edge comprised of single and branching filaments (Figure 2, A and B). Consequently, Jeg-3 cells, which typically form monolayers, instead form 3-dimensional stacks on top of their neighboring cells when ARHGAP18 signaling is disrupted (Figure 4, D and E). In endothelial cells, ARHGAP18 has been reported to localize microtubules and plays a role in maintaining proper microtubule stability (Lovelace *et al.*, 2017).In our epithelial cell culture models and WT mouse intestine, we have been unable to detect ARHGAP18 at microtubules, suggesting ARHGAP18 may have additional functions in various cell types.

Collectively, our results support a model where loss of ARHGAP18 depolarizes cells by dysregulating apical, junctional, and basal membrane identification through ERMs, Merlin, and YAP, respectively. The exact molecular interactors responsible for the signaling cascade that results in pMLC activation in the overexpression of ARHGAP18 will require future characterization. Additional investigation of the molecular details of the ARHGAP18/YAP binding complex will elucidate the mechanism that ARHGAP18 uses to coordinate RhoA signaling with the Hippo pathway to define cell polarity and morphology.

## METHODS

### Reagents and cDNAs

Anti-ezrin antibody (CPTC-ezrin-1 supernatant concentrate obtained from the Developmental Studies Hybridoma Bank; catalogue no. CPTC-Ezrin-1; Research Resource Identification AB_2100318) was used 1:100 for immunofluorescence. Mouse anti-Flag (Sigma-Aldrich; catalogue no. F1804) was used at 1:250 for immunofluorescence, and 1:5,000 for Western blot, and mouse anti-tubulin (Sigma-Aldrich; catalogue no. T5168) was used at 1:5,000 for Western blot. Anti-MLC2 (catalogue no. 3672) and anti–phospho-MLC2 (phospho-Thr18/Ser19; catalogue no. 3674) were purchased from Cell Signaling Technology and used at 1:500 in Western blots. Anti–nonmuscle myo-2B from BioLegend (catalogue no. 909902) and non-muscle myo-2A (BioLegend; catalogue no. 909802) were used at 1:100 in both Western blots and immunofluorescence. Colocalization analysis of basal contractile bundles was performed by overlaying the actin and NM-Myo-2A/B immunofluorescent images in ImageJ, then counting the number of large (1 µm or longer) actin bundles containing regular repeats of NM-Myo-2B along the length of the bundle using the plugin MTrackJ. Bundles near the junctions, edges of cells, or in overlapping asters were excluded as they could not be accurately counted or distinguished from junctional structures. The rabbit ARHGAP18 antibody was produced and characterized previously (Lombardo *et al.*, 2024) and used at 1:500 for Western blotting. Paxillin from Abcam Antibodies (catalogue no. 32084) was used at 1:100 for immunofluorescence. Rabbit anti-YAP/TAZ (Cell Signaling Technologies #14074) and pYAP (s125; Cell Signaling Technologies #13008) were used at 1:100 for immunofluorescence and 1:1000 for Western blot. For actin staining, Alexa Fluor 647 phalloidin (Invitrogen; catalogue no. A30107) was used at 1:250.

Human ARHGAP18 constructs were produced using polymerase chain reaction (PCR) with New England Biolabs (NEB) Phusion High-Fidelity PCR Kit (Catalogue # E0553L). cDNA of ARHGAP18 was obtained from a construct originally derived from the Harvard Plasma Database (ID # HsCD00379004). NEB Monarch PCR & DNA Cleanup Kits (Catalogue # T1030S) and Thermo Scientific GeneJET Gel Extraction Kits (Catalogue # K0692) were used to purify PCR and Gibson Assembly products. DNA products were cloned into the mammalian expression vector PQCXIP using NEB Gibson Assembly cloning Kit (Catalogue # E5510S). Transformations were done into OneShot TOP10 bacteria (Thermo Fisher; Catalogue # C404010), selected using ampicillin resistance, and then sequenced for verification. GFP-merlin was a gift from A. Bretscher and produced previously (Sher *et al.*, 2012). The GAP-flag-EBP50(tail) vector was generated by PCR amplification of the EBP50 tail sequence from the GFP-EBP50 construct, using the methods described in Lombardo *et al*. (2024). Gibson assembly was used to insert the EBP50 tail construct into the GAP-flag vector.

### Western blotting

Western blot analysis of cell lysates was done using 4%–12% Thermo Fisher Bolt SDS–PAGE gels (Catalogue # NW04120). Gels were transferred to a polyvinylidene difluoride (PVDF) membrane using a BioRad Transblot Turbo (Catalogue # 1704150) and blocked with 5% milk in Tris Buffered Saline (TBS) + 0.5% Tween-20 (TBST). For detecting MLC and pMLC, 0.2-µm pore size PVDF (EMD Millipore; Immobilon-PSQ) was used. Quantification of Western blots was performed on the same blot using paired mouse and rabbit antibodies and Alexa-680 nm anti-mouse with Alexa-800 nm anti-rabbit whenever possible. For blots that required quantification of antibodies that were only rabbit primaries (e.g., pMLC/MLC antibodies listed above), samples were loaded onto a single gel and transferred onto a single membrane at the same time. After transfer, the membrane was cut in half, and subsequent steps were done in parallel. All quantified blots were checked for equal loading using either anti-tubulin as a housekeeping protein or total protein as detected by Coomassie staining. Primary antibodies were incubated with the membrane in TBST supplemented with 5% BSA either for 1 h at room temperature or overnight at 4°C. Bands were detected with infrared fluorescent secondary antibodies (Invitrogen or LI-COR Biosciences; Catalogue nos. 926-32221 and 926-32210). Membranes were imaged using a Bio-Rad MP ChemiDoc. The ImageJ intensity profile built-in toolset was used for western blotting quantification, which was then averaged, normalized, and plotted in Microsoft Excel.

### Immunofluorescent imaging

Room temperature (23°C) fixed cell confocal imaging was done using a spinning-disk (Yokogawa CSU-X1; Intelligent Imaging Innovations) Leica DMi600B microscope with a spherical aberration correction device and either a ×100/1.46 NA Leica objective. Hamamatsu ORCA-Flash 4.0 camera metal-oxide semiconductor device was used to capture images, and Z-slices of acquired images were assembled using SlideBook 6 software (Intelligent Imaging Innovations). SORA imaging was performed on a spinning-disk (Yokogawa CSU-W1) Intelligent Imaging Innovations Marianas microscope with a x63/1.4NA Zeiss objective and Hamamatsu ORCA Quest camera. Maximum-intensity projections were assembled in SlideBook 6 or ImageJ and exported to Adobe Illustrator for editing. Vertical expansion of side projections was used to increase visual clarity of apical/basal localization. Widefield microscopy was performed using an inverted Leica DMi8 widefield microscope equipped with a Leica ×100 NA air objective, a Leica DFC 9000 GTC camera, Leica Application Suite X, and Leica adaptive focus control.

Cells for immunofluorescence confocal or SORA confocal imaging were plated onto Warner Instruments high tolerance, 1.5 glass coverslips (Catalogue # 64-0734) and allowed to grow to the appropriate confluency for 24–48 h. Cells were fixed by washing cells with room temperature PBS pH 7.4 three times, followed by a 10-min incubation in 10% Paraformaldehyde (PFA) in PBS pH 7.4. Cells were washed 3x in PBS and permeabilized using 0.2% Triton-X in PBS for 5 min at room temperature. After washing with PBS three times, cells were blocked using 2% FBS fetal bovine serum (FBS; Thermo Fisher Scientific; Catalogue # 26140079) in PBS at room temperature for 10 min. Primary antibodies were diluted into 2% FBS in PBS as listed in the reagents section and incubated for 1 h. at room temperature, followed by washing in PBS and incubation in secondary antibody with Alexa-dye phalloidin (Invitrogen; Catalogue no. A30107) at 1:250 and DAPI at 1:10000 for 1 h. at room temperature in 2% FBS in PBS. Cells were washed 3x in PBS at room temperature, then mounted onto Fisher Scientific Premium Plain Glass Microscope Slides (Catalogue # 12-544-4) using Invitrogen ProLong Diamond Antifade Mountant (Catalogue # P36961).

### Mouse intestine immunofluorescence

Ten-week-old mice were anesthetized with Isoflurane and perfused with 25 ml of cold 1X PBS followed by 25 ml of 4% PFA. Small Intestines were collected and flushed with 40 ml of cold 1X PBS and cut longitudinally to expose the luminal surface. Intestines were then rolled longitudinally with the luminal surface facing inward to create a Swiss roll and post-fixed with 4% PFA at 4°C for 24 h. Intestines were then cryoprotected with 30% sucrose for 72 h at 4°C and embedded in Optimal Cutting Temperature Media. Cryosections were generated at a thickness of 15 mm with a Microm HM 505 N cryostat (Thermo Fischer Scientific, Waltham, MA) and frozen onto Superfrost Plus microscope slides (Fisher Scientific). Samples were washed with 1x PBS and permeabilized/blocked in 1X PBS-T (2% Triton-X) containing 5% Normal Horse Serum for 1 h at room temperature (RT). Samples were then stained overnight at 4°C in 1x PBS containing 2% normal horse serum and primary antibodies at the following dilutions: Rabbit anti-ARHGAP18 (1:10), Mouse anti-Ezrin (1:100). Samples were then washed with 1X PBS and incubated for 1 h at RT in 1x PBS containing 2% normal horse serum and secondary antibodies at the following dilutions: Donkey anti-rabbit Alexa Fluor 568 (1:500; Invitrogen; Catalogue no. A10042), goat anti-mouse Alexa Fluor 488 (1:500; Invitrogen; Catalogue no. A32723), phalloidin-Alexa-647 (1:500; Invitrogen; Catalogue no. A30107), and Hoechst 33342 (1:3000; AnaSpec, 83218). Samples were mounted with Fluoromount-G (Invitrogen, 00-4958-02). Images were captured with a Yokogawa CSU-X1 spinning-disk Leica DMi600B microscope (Intelligent Imaging Innovations, Tokyo, Japan). All animal protocols and procedures were approved by the SUNY Buffalo Animal Care and Use Committee under IACUC protocol BCH08076N.

### Immobilized ARHGAP18 pulldowns

To determine the interaction between ARHGAP18 and Merlin or YAP, pulldown assays were performed using cell lysate from Jeg-3 WT cells. Purified human ARHGAP18 was produced by bacterial expression of an N-terminal-SUMO-HIS-tagged protein purified using a NiNTA resin as described in (Lombardo *et al.*, 2024). Transfections for GFP Merlin were performed using PEI MAX polyethylenimine reagent (Polysciences; Catalogue # 24765). Cells were harvested with lysis buffer (25 mM Tris, 5% glycerol, 150 mM NaCl, 50 mM NaF, 0.1 mM Na3VO4, 10 mM βGP, 0.2% Triton X-100, 250 mM calyculin A, 1 mM DTT, 1× cOmplete Protease Inhibitor Cocktail [Roche; Catalogue # 11836153001]) by scraping. Lysates were then sonicated and centrifuged at 8000 × g for 10 min at 4°C. Before incubating with the cell lysates, SUMO-ARHGAP18 NiNTA beads were equilibrated and washed in lysis buffer. The sample of the supernatant was taken for input, then the rest was added to the SUMO-ARHGAP18 NiNTA beads and nutated for 3 h at 4°C. After incubation, the beads were pelleted and washed four times with a 2 min incubation before boiling in 40 µl 2× Laemmli sample buffer.

### RNA-Seq sample preparation

Cells were plated in 100 mm Corning 100 × 20 mm Petri-style TC-treated culture dishes (Catalogue# 430167) and allowed to grow to 80% confluency. Cells were plated at 10 am on day 0, media was exchanged at 10 am on day 1, and cells were collected and pelleted at 10 am on day 2 over a total of 48 h to maintain environmental consistency. Pelleting of cells was performed by washing the cells three times in PBS, then adding 0.05% trypsin (Thermo Fisher Scientific; Catalogue # 25300054) and incubating at 37°C and 5% CO2 for 5 min. Once detached cells were washed into MEM media (Thermo Fisher Scientific; Catalogue# 10370088), then the media was exchanged for ice-cold PBS. Cells were pelleted at 1000x g and stored at −80°C until they were lysed for RNA extraction. Total RNA was extracted at the University at Buffalo Core Genomics Facility using TRIzol reagent (Catalogue no. 15596018, Invitrogen). It was then purified with a RNeasy kit (Catalogue no. 74106, Qiagen). Libraries were prepared using the Illumina TruSeq Stranded total RNA kit and the Ribo-Zero plus rRNA depletion kit. The sequencing was performed on an Illumina HiSeq 4000 PE100 sequencer. The nf-core/rnaseq Version 3.18.0 bioinformatics pipeline was used to analyze RNA sequencing data, with alignment and quantification performed using STAR (Dobin *et al.*, 2013) followed by Salmon (Patro *et al.*, 2017).

### Bioinformatic analysis

Raw gene counts were analyzed using the DESeq2 package in R to identify differentially expressed genes (DEGs) between WT-Jeg-3 and ARHGAP18 KO conditions. Genes were classified as DEGs if the following filtering criteria were met: false-discovery rate (FDR) adjusted *p*-value of <0.05 and absolute log_2_(fold-change) of > 0.5. Sample distribution was visualized with the Principal Component Analysis (PCA) plot generated by the ggplot2 package in R using a normalized count dataset. Python's Bioinfokit was used to create the volcano plot.

Functional enrichment analysis was performed using the g: GOSt function on the gProfiler web server (https://biit.cs.ut.ee/gprofiler/gost) with the identified DEGs. The significance threshold was set to the FDR value, and significant Gene Ontology (GO) terms in molecular functions, biological pathways, and cellular components categories were defined by an adjusted *p*-value of < 0.05. The top 15 GO terms in each category were visualized using bubble plots generated by the SRplot online server.

To visualize expression patterns across samples, a clustered heatmap was generated with the SRplot online server using log_2_-transformed normalized gene expression values. Specifically, hierarchical clustering was performed on both genes (rows) and samples (columns) using Euclidean distance and complete linkage cluster methods. Genes visualized were DEGs annotated in the GO term.

Ingenuity pathway analysis (IPA, QIAGEN) was used to perform network analysis on 889 DEGs identified in this dataset. A core analysis was run on this dataset, which produced information on various mechanistic pathways and enriched function-based literature gathered in the IPA knowledge base. Specifically, we used the “My Pathway” function to generate predicted networks between ARHGAP18 and some genes of interest. The “Molecular Activity Predictor” tool was used to elucidate predicted activation states of molecules and interactions dependent on ARHGAP18.

### RT-qPCR

Total RNA was reverse transcribed and analyzed by RT-qPCR as previously described (Klein *et al.*, 2007). Briefly, RNA samples were diluted to 50 ng/ml with molecular-grade biology water and used for reverse transcription with Applied Biosystem reagents (1x buffer, MgCl2, dNTP, random hexamers, RNase inhibitor, and Multiscribe Reverse Transcriptase) in a total volume of 20 µl. The following PCR reactions contain 2 µl of the complementary DNA mix, TaqMan Universal PCR mix, forward and reverse primers, and targeted probes. TaqMan probes were used for CORO1A (Invitrogen, Hs00200039_m1) and GAPDH (Invitrogen, Hs02786624_g1). The PCR reaction contains 40 cycles of denaturation (5 min at 95°C) and annealing/extension (30 s at 60°C). The relative change in mRNA expression for the target gene was calculated using the comparative threshold cycle method, with GAPDH serving as the reference gene. RNAseq data were uploaded to GEO accession GSE310589.

### Cell culture and expression vectors

Jeg-3 cells were purchased from ATCC.org (Catalogue # Htb-36) and maintained in a humidified incubator at 37°C and 5% CO2. Media for Jeg-3 cells used in 1× MEM (Thermo Fisher Scientific; Catalogue # 10370088) with penicillin/streptomycin (Thermo Fisher Scientific; Catalogue # 15070063), 10% fetal bovine serum (FBS; Thermo Fisher Scientific; Catalogue # 26140079), and GlutaMAX (Thermo Fisher Scientific; Catalogue # 35050061). Corning 100 × 20 mm Petri-style TC-treated culture dishes (Catalogue # 430167) were used for maintaining cultures. Cell cultures for experiments were maintained at 70%–80% confluency. For focal adhesion experiments, the cell cultures were maintained at 50%–60% confluency. Transient transfections used a PEI MAX polyethylenimine reagent (Polysciences; Catalogue # 24765). All cell lines were checked for mycoplasma in regular intervals using DAPI staining. ARHGAP18 knockout cells were created and characterized (Lombardo *et al.*, 2024). In summary of these methods, multiple CRISPR single-guide RNAs against the sequence 5′-CAGCGGCAAGGACCAGACCG-3′ or 5′-CAGCGGCAAGGACCAGACCG-3′ were cloned into puromycin-resistant pLenti-CRISPRV2 (Addgene; Catalogue # 49535), which was transfected into 293TN cells with psPAX3 and pCMV-VSV-G (a gift from Jan Lammerding, Weill Institute for Cell and Molecular Biology, Cornell University, Ithaca, NY) for 48–72 h. Jeg-3 cells were then transduced with virus supplemented with Polybrene to 8 µg/ml twice a day for 2 d, followed by puromycin selection at 2 µg/ml. Mixed populations of selected cells were single-cell sorted and then screened by western blotting. ARHGAP18 expression vectors were created and described in (Lombardo *et al.*, 2024).

### Super-resolution STORM

Jeg-3 cells were plated on 35 mm 1.5 high precision glass bottom MatTek dishes (Catalogue # P35G-0.170-14-C) and fixed and permeabilized using 2 mls of 0.3% glutaraldehyde and 0.25% Triton X-100 in cytoskeleton buffer (10 mM MES pH 6.1, 150 mM NaCl, 5 mM EGTA, 5 mM glucose, and 5 mM MgCl_2_) for 1–2 min at room temperature. Initial fixation was followed by 2% glutaraldehyde in cytoskeletal buffer for 10 min at room temperature. The sample was then treated with 1ml 0.1% NaBH4 in Phosphate Buffered Saline (PBS) at pH 7.4 for 7 min to reduce background fluorescence. Actin was stained with phalloidin at a dilution of 1:250 overnight at 4°C. Slides were washed into 50 mM Tris, pH 8.0, 10% glucose, 10 mM NaCl, 0.5 mg/ml Glucose oxidase, 0.04 mg/ml catalase, with 10% cysteamine by weight, and 1.5% 2-Mercaptoethanol (BME) by weight. Stochastic Optical Reconstruction Microscopy (STORM; Rust *et al.*, 2006) was performed using a Zeiss Elyra super-resolution inverted Axio Observer.Z1 microscope provided by Cornell University's Institute of Biotechnology Imaging Facility. Lasers emitting 405 and 640 nm wavelengths through a 100x/1.46 NA oil objective captured images on a pco.edge 5.5m camera. Exposure time ranged from 50 to 400 ms and was dependent on the sample and channel to optimize STORM reconstruction. A minimum of 100,000 images were used for each STORM processing using the ImageJ ThunderSTORM toolset (Ovesny *et al.*, 2014) or the Zeiss Zen built-in STORM reconstruction toolset, and adjusting the filtering conditions to maximize the signal-to-noise of single actin filaments in reconstructions.

### Statistical methods

Statistical comparisons were performed in Microsoft Excel. The type of statistical tests utilized, the configuration of error bars, and the number of independent data points (*n*) are detailed in the figure legends respective to the data tested. Nonparametric or parametric testing was justified through the assumption of the tested data having a normal distribution or not. Analyses were also done using GraphPad Prism to produce a graphical representation of the amount of phosphorylated YAP over the total expression of YAP with the test and *n* listed in the figure legend.

## Supplementary Material



## Abbreviations used:

ERM: ezrin radixin moesin
GAP: GTPase-activating protein
LOK: lymphocyte-oriented kinase
MLC: myosin light chain
NM-Myo-2: non-muscle myosin 2
SORA: super resolution by optical pixel reassignment
YAP: yes-associated protein.
