# Supplementary material for: ARHGAP18 complexes with both YAP and Merlin and is required for basal actin bundles
Source: Mol Biol Cell. 2026 Jun 30;37(7):ar75. doi: 10.1091/mbc.E26-03-0143 (PMC13329807; doi:10.1091/mbc.E26-03-0143)

Supplemental Materials

Molecular Biology of the Cell

Murray *et al*.

A

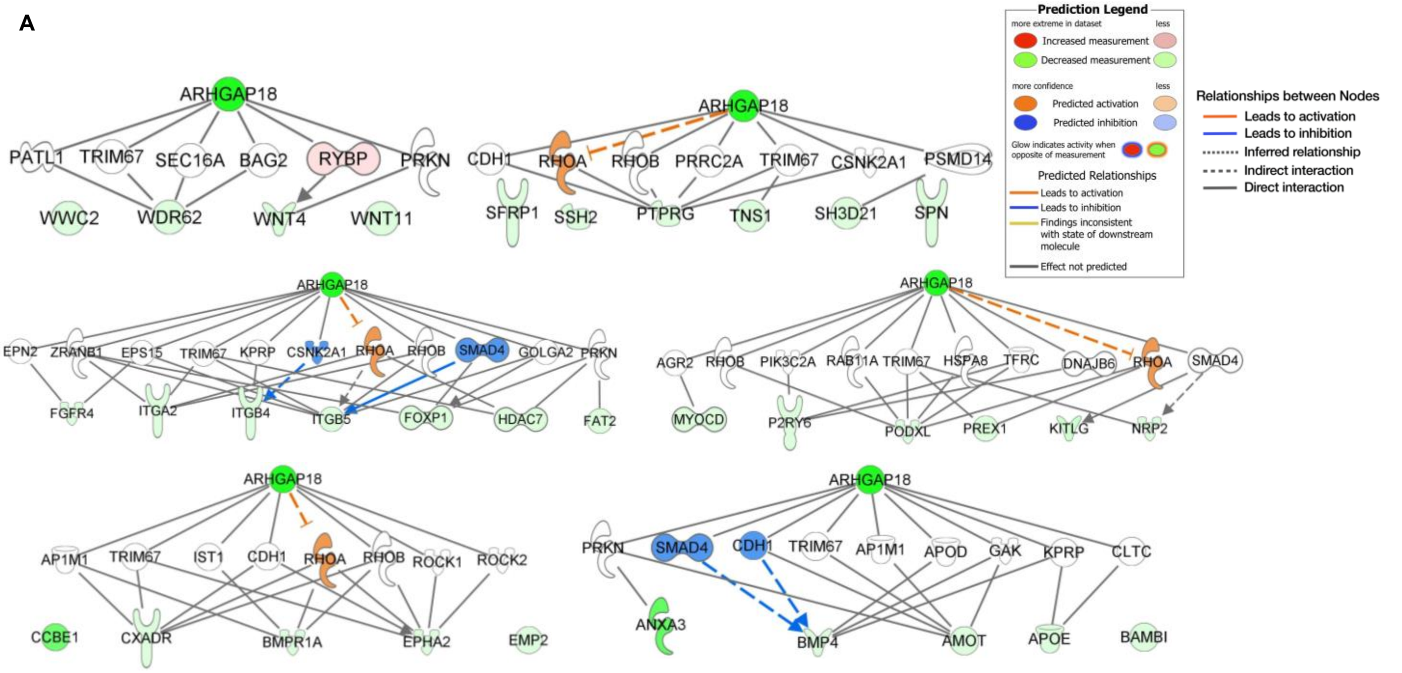

B

Upstream regulator predicted to be involved due to expressional changes seen in the dataset (predicted inhibition)

Upstream Regulator	Molecule Type	Predicted Activation State	Activation z-score	p-value of overlap	Target Molecules in Dataset
FOXA1	transcription regulator	Inhibited	-3.116	5.59E-11	↑ANXA1, ↓AQP1, ↑ARHGEF9, ↑BHLHE40, ↑BNIP3L, ↑B... ...all 30
SFTPA1	other	Inhibited	-3.000	3.63E-04	↓AMOT, ↑CRLF1, ↑ECM1, ↓GABRP, ↑PLIN2, ↑SERPINE1, ...all 9
N-[N-(3,5-difluorophenacetyl-L-Ala)]-S	chemical - protease inhibitor	Inhibited	-2.944	1.91E-03	↓CD24, ↑FSTL3, ↑HAPLN1, ↑HEY1, ↑JAG1, ↑LOC10272... ...all 12
miR-141-3p (and other miRNAs w/see	mature microRNA	Inhibited	-2.612	1.09E-04	↑CD274, ↑JAG1, ↑PITX1, ↑PMAIP1, ↓RHPN2, ↑RNF128, ...all 10
NR0B2	ligand-dependent nuclear receptor	Inhibited	-2.599	1.05E-01	↑CARD11, ↓CYP7B1, ↑FGFR1, ↑MAP3K8, ↑PIK3C2G, ↑S... ...all 8
ETV6-RUNX1	fusion gene/product	Inhibited	-2.571	2.25E-03	↓ABLIM1, ↓ABR, ↑ANTXR2, ↑ARHGAP24, ↑CARD11, ↑... ...all 19
26S PROTEASOME (complex)	complex	Inhibited	-2.530	1.28E-02	↑ATP6V1H, ↑CTSB, ↑CTSF, ↓FOXO1, ↑HSPB8, ↑NOTCH1 ...all 10
CAPN6	peptidase	Inhibited	-2.530	3.76E-04	↓BMP4, ↓BMPR1A, ↓FZD7, ↓ID1, ↑SERPINE1, ↓TCF7, ↑... ...all 10
EGLN (family)	group	Inhibited	-2.459	9.06E-04	↑BNIP3, ↓DMKN, ↑FSTL3, ↑MAP3K8, ↓MYOCD, ↑PDG... ...all 13
salirasib	chemical drug	Inhibited	-2.433	6.87E-03	↑BHLHE40, ↑BNIP3, ↑ENO2, ↑PFKFB4, ↑SERPINE1, ↑STC1 ...all 6
CCN5	growth factor	Inhibited	-2.401	4.44E-03	↓CD24, ↑PROCR, ↑SERPINE1, ↑TGFB1, ↑TWIST1, ↑ZNF4... ...all 6
miR-155-5p (miRNAs w/seed UAAUGC	mature microRNA	Inhibited	-2.359	7.68E-02	↑AMIGO2, ↑MYO10, ↑MYO1E, ↑PICALM, ↑PMAIP1, ↓P... ...all 9
ALPHA CATENIN (family)	group	Inhibited	-2.343	8.80E-03	↑CTSB, ↑ELK3, ↓IGF2, ↑LYN, ↑SGK1, ↑SNAI1, ↑TNFAIP3, ...all 9
ESR1	ligand-dependent nuclear receptor	Inhibited	-2.254	7.16E-10	↓ABCA4, ↓ABLIM1, ↑ADCY1, ↑ALCAM, ↓ANGPTL2, ↓A... ...all 96
ibrutinib	chemical drug	Inhibited	-2.236	4.51E-01	↓BMP4, ↑COX6C, ↓MGLL, ↑RIPK2, ↑WDR74 ...all 5
CELF1	translation regulator	Inhibited	-2.236	6.33E-03	↓ADGRL2, ↓DMPK, ↓DNMBP, ↑JAG1, ↓PXDN, ↑WFS1 ...all 6
OVOL2	transcription regulator	Inhibited	-2.224	1.42E-02	↑ARHGAP31, ↑FAM13A, ↑NR2F1, ↑SDCBP, ↑WIPF1 ...all 5
TNFSF12	cytokine	Inhibited	-2.216	3.65E-01	↑HEY1, ↑HEYL, ↓ID1, ↑MYOD1, ↑NOTCH1 ...all 5
mir-34 (includes others)	microRNA	Inhibited	-2.207	2.38E-02	↑BNIP3L, ↑FGFR1, ↑HEY1, ↑KLF10, ↓LIN28A, ↑NOTCH1, ...all 8
GIP	other	Inhibited	-2.200	2.90E-02	↑IKBKB, ↓MX1, ↑SERPINE1, ↓TCF7, ↑TNFRSF1B ...all 5
DCAF1	kinase	Inhibited	-2.177	5.57E-03	↑CD274, ↑KLF10, ↑PMAIP1, ↑SOCS2, ↑TNFSF10 ...all 5
PDLIM2	other	Inhibited	-2.111	8.22E-05	↑CRISPLD2, ↓DPF3, ↑MYL9, ↑NR2F1, ↑PGCKA1, ↑RAS... ...all 11
iron	chemical - endogenous mammalian	Inhibited	-2.085	2.07E-02	↑GADD45G, ↓HMOX1, ↓ID1, ↑NDRG1, ↑SLC40A1 ...all 5
IL10RA	transmembrane receptor	Inhibited	-2.065	2.02E-03	↓ADGRL2, ↓ALDH2, ↑ASS1, ↓BMP4, ↑BNIP3, ↑CCR7, ↓... ...all 19
mifepristone	chemical drug	Inhibited	-2.022	2.03E-04	↓ACAN, ↑ANXA1, ↓APOE, ↓AQP1, ↑CAV1, ↓CD24, ↑C... ...all 23
N-[(2Z)-3-(4,5-dihydro-1,3-thiazol-2-yl)	chemical reagent	Inhibited	-2.008	2.68E-03	↑CYP19A1, ↑ELOVL3, ↑LYPD3, ↑NR4A3, ↑SERPINE1, ↑S... ...all 8
HSP70 (family)	group	Inhibited	-2.000	8.08E-02	↓ACSL3, ↑GBP1, ↑MAPT, ↑SAT1, ↑SLC40A1 ...all 5
LAS1L	other	Inhibited	-2.000	9.85E-02	↑ASS1, ↑RN7SK, ↑SCARNA13, ↑SMPDL3B ...all 4
SKIC2	enzyme	Inhibited	-2.000	4.28E-02	↑BHLHE40, ↑CTH, ↑DDIT4, ↑STC1 ...all 4
ILKAP	phosphatase	Inhibited	-2.000	3.89E-03	↑CCR7, ↓FOXO1, ↑NDRG1, ↑NR4A3 ...all 4

C

Upstream regulator predicted to be involved due to expressional changes seen in the dataset (predicted activation)

Upstream Regulator	Molecule Type	Predicted Activation State	Activation z-score	p-value of overlap	Target Molecules in Dataset
TP53	transcription regulator	Activated	4.101	8.87E-10	↓ACSL3, ↓ACSL6, ↑AGO2, ↑ANXA1, ↓ANXA3, ↑APOBE.....all 125
dimethyl n-oxalyl-glycine	chemical reagent	Activated	3.969	4.74E-10	↑BNIP3, ↑BNIP3L, ↑BTG1, ↑CAV1, ↑CORO1A, ↑DDIT4, ↑.. ...all 28
BHLHE40	transcription regulator	Activated	3.797	2.03E-05	↑ANXA1, ↑BHLHE40, ↑BHLHE41, ↑BTG1, ↑CAV1, ↑CCR7, ...all 25
CD3 (complex)	complex	Activated	3.557	1.13E-05	↑ANXA1, ↓ATP11B, ↓BAMBI, ↑BTG1, ↑CD274, ↑CD53, ↑.. ...all 39
IL13	cytokine	Activated	3.394	2.35E-04	↑AVPI1, ↑CISH, ↑CTSB, ↑CTSH, ↑CXCR2, ↑ELP3, ↑ERBB4, ...all 30
CALCA	other	Activated	3.301	3.38E-03	↑GAST, ↓HMOX1, ↑IL2RB, ↑JAG1, ↑PLEKHA6, ↑PLXDC2, ...all 13
doxorubicin	chemical drug	Activated	3.300	1.82E-03	↑ANXA1, ↑BHLHE40, ↓BMP4, ↑BNIP3L, ↑BTG1, ↑CD274, ...all 40
ZBTB16	transcription regulator	Activated	3.293	5.87E-02	↓AGPAT4, ↓CD24, ↑CD274, ↑CSGALNACT1, ↑DDIT4, ↑F... ...all 11
IL6	cytokine	Activated	3.199	4.92E-05	↓ADGRL2, ↑ANXA1, ↓APOE, ↓BAMBI, ↑BHLHE40, ↑CCR7 ...all 47
lipopolysaccharide	chemical drug	Activated	3.180	4.76E-08	↓ACAN, ↑ALCAM, ↓ALDH2, ↓ANGPTL2, ↑ANXA1, ↓A... ...all 138
PIWIL4	other	Activated	3.162	7.04E-03	↑ADSS1, ↑BTG1, ↑ELK3, ↑FYB1, ↑MFGE8, ↑NDRG1, ↑S... ...all 10
PTGS2	enzyme	Activated	3.076	5.53E-03	↑ADGRL3, ↑ANXA1, ↓AQP1, ↑CCR7, ↑CD274, ↑CXCR2, ...all 15
CSF2	cytokine	Activated	2.986	1.47E-02	↑ANXA1, ↓BMPR1A, ↑CARD11, ↑CCR7, ↓CD24, ↑CD274, ...all 25
etoposide	chemical drug	Activated	2.914	2.94E-02	↑CD274, ↑CLCA2, ↑CSF2RB, ↑DDIT4, ↑GADD45G, ↑GBP2, ...all 15
olaparib	chemical drug	Activated	2.897	1.24E-04	↑BHLHE40, ↑BOK, ↑BTG1, ↑GADD45G, ↑GBP2, ↑GDF15, ...all 14
PDGF-BB (complex)	complex	Activated	2.830	1.15E-04	↑BHLHE40, ↓BMP4, ↑CAV2, ↑CTH, ↓F3, ↑FGFR1, ↑GBP2, ...all 24
PD173074	chemical reagent	Activated	2.828	6.74E-11	↑CCR7, ↑CDH5, ↑CGA, ↑CYP19A1, ↑ERVFRD-1, ↓F3, ↓... ...all 18
CD28	transmembrane receptor	Activated	2.828	1.99E-05	↑ANXA1, ↓ATP11B, ↑BHLHE40, ↑BTG1, ↑CD274, ↑CISH, ...all 26
EGF	growth factor	Activated	2.807	6.40E-06	↓ACSL3, ↑ALCAM, ↑CAV1, ↑CD274, ↑CDH5, ↑CGA, ↑CI... ...all 44
NLRP7	other	Activated	2.795	4.47E-07	↑APOBEC3C, ↑CD274, ↓FBXO38, ↑FXYD3, ↓MAGEA4, ↑N... ...all 8
tetradecanoylphorbol acetate	chemical drug	Activated	2.784	3.84E-09	↓ACAN, ↑ADAM9, ↓ADCY3, ↑ALCAM, ↑AMIGO2, ↑ANX... ...all 77
STAT4	transcription regulator	Activated	2.783	1.41E-05	↑BHLHE40, ↑ENO2, ↑FYB1, ↓FZD7, ↑GADD45G, ↑MAG... ...all 20
AHR	ligand-dependent nuclear receptor	Activated	2.762	4.31E-05	↑ADAM9, ↑AHRR, ↓ALDH2, ↑AMIGO2, ↓BAMBI, ↑CAD... ...all 36
3,5-dihydroxyphenylglycine	chemical reagent	Activated	2.714	3.23E-03	↑BCAS1, ↓CACNA1D, ↑ELAVL2, ↑FABP7, ↓FAT2, ↓L1CAM, ...all 11
LEF1	transcription regulator	Activated	2.688	7.20E-04	↓ACAN, ↑ANXA1, ↑CARD11, ↑CAV1, ↓CD24, ↑CD274, ↑.. ...all 20
IL1B	cytokine	Activated	2.659	5.79E-10	↓ACAN, ↓ANGPTL2, ↑ANXA1, ↓APOE, ↑ASS1, ↓BACE1, ...all 80
enzalutamide	chemical drug	Activated	2.630	7.35E-03	↑BOK, ↓CYP4V2, ↑ENO2, ↑GADD45G, ↑PPP1R13B, ↑TGF... ...all 8
IL4	cytokine	Activated	2.576	8.06E-08	↓ABLIM1, ↓ACSL6, ↑ADCY1, ↓AGPAT4, ↓ALDH2, ↑ANX... ...all 74
pirinixic acid	chemical toxicant	Activated	2.547	4.69E-03	↑APOA2, ↓APOE, ↑BNIP3, ↓CD24, ↑CGA, ↑CTSB, ↓CYP... ...all 24
STAT3	transcription regulator	Activated	2.526	3.31E-09	↓ANGPTL2, ↑BNIP3L, ↑CASC9, ↑CCR7, ↑CD274, ↑CDH5, ...all 58
NR3C2	ligand-dependent nuclear receptor	Activated	2.520	5.53E-04	↑APOC1, ↓APOE, ↓BMP4, ↑CACNA1H, ↑CXCR2, ↑DDIT4, ...all 15

Upstream Regulator	Molecule Type	Predicted Activation State	Activation z-score	p-value of overlap	Target Molecules in Dataset
ALKBH5	enzyme	Activated	2.190	1.56E-02	↑BNIP3, ↑CAV1, ↑FABP5, ↑FGFR1, ↓FOXO1, ↓IGF2, ↑ND... ...all 9
KLF15	transcription regulator	Activated	2.187	9.80E-02	↑DDIT4, ↑DECR1, ↑FABP5, ↑IRX3, ↑SLC27A1, ↑TNFSF10 ...all 6
methylnitronitrosoguanidine	chemical toxicant	Activated	2.186	3.09E-03	↑BTG1, ↑GDF15, ↑KLF10, ↑SERPINE1, ↑TNFSF10 ...all 5
D-glucose	chemical - endogenous mammalian	Activated	2.176	7.05E-05	↓ACAN, ↑ANXA1, ↓ANXA3, ↓AQP1, ↑ASS1, ↓BMP4, ↓... ...all 45
IL5	cytokine	Activated	2.174	9.10E-04	↑ASS1, ↑BNIP3, ↑BNIP3L, ↑CCR7, ↓CD24, ↑CISH, ↑CSF... ...all 19
glucocorticoid	chemical drug	Activated	2.162	4.41E-02	↑ANXA1, ↑CGA, ↑CYP19A1, ↓GABRP, ↑GBP1, ↓GPR158, ...all 15
ADIPOQ	other	Activated	2.160	7.17E-05	↓ANGPTL2, ↑APOA2, ↑APOC1, ↑CGA, ↓CHIC1, ↑CYP19A1 ...all 22
SREBF1	transcription regulator	Activated	2.146	2.87E-03	↑APOA2, ↑BHLHE40, ↑BHLHE41, ↑CTH, ↓CXADR, ↓CYP... ...all 18
IL2	cytokine	Activated	2.141	5.44E-05	↓ACSL3, ↓ACSL6, ↓ADCY3, ↑ANXA1, ↓AUTS2, ↑BHLHE40 ...all 43
TYA-018	chemical reagent	Activated	2.138	2.57E-03	↓ACSL3, ↑BNIP3, ↑CAV1, ↓CHMP6, ↑DECR1, ↑GGH, ↑... ...all 14
IL15	cytokine	Activated	2.127	1.00E-04	↑ANXA1, ↓BAMBI, ↑BTG1, ↑CD274, ↑CD53, ↑CISH, ↑C... ...all 31
cantharidin	chemical drug	Activated	2.121	1.15E-03	↓ALDH3A2, ↑BHLHE40, ↑BHLHE41, ↑CYP19A1, ↑ELK3, ↑.. ...all 26
CD38	enzyme	Activated	2.111	5.35E-04	↑ASS1, ↑BNIP3, ↑BNIP3L, ↑CISH, ↑CSF2RB, ↑FAM162A, ...all 14
NEDD9	other	Activated	2.111	4.95E-05	↑BHLHE40, ↑BNIP3, ↑DDIT4, ↑GDF15, ↓ITGA2, ↑PLIN2, ...all 11
IL17A	cytokine	Activated	2.101	1.37E-02	↓ACAN, ↑CD274, ↑COTL1, ↓CYP7B1, ↓HMOX1, ↑HSPB8, ...all 23
semaxinib	chemical drug	Activated	2.093	1.20E-07	↑AHRR, ↑CARD11, ↑CCR7, ↓CD24, ↑CSF2RB, ↑CYP19A1, ...all 18
lactic acid	chemical - endogenous mammalian	Activated	2.085	2.35E-03	↓AGRN, ↑ARRDC3, ↑CACNG4, ↑CD274, ↓CDH3, ↑CPLX1, ...all 16
STAT6	transcription regulator	Activated	2.060	1.91E-02	↓ABLIM1, ↓ALDH2, ↑ANXA1, ↓APOE, ↑ASS1, ↑CCR7, ↓... ...all 24
ethanol	chemical - endogenous mammalian	Activated	2.056	3.83E-01	↓BMP4, ↓CYP7B1, ↑ELOVL3, ↑FABP5, ↑FGFR1, ↑GSR, ↑... ...all 18
poly rI:rC-RNA	biologic drug	Activated	2.035	7.78E-04	↓ACSL3, ↓AGRN, ↑ANXA1, ↑APOBEC3C, ↓AUTS2, ↑BNI... ...all 44
JAG1	growth factor	Activated	2.030	5.63E-05	↑CYP19A1, ↑GDF15, ↑GPX3, ↑HEY1, ↑HEYL, ↑JAG1, ↑R... ...all 10
5-fluorouracil	chemical drug	Activated	2.009	1.85E-03	↑BNIP3, ↑BNIP3L, ↓CD24, ↑COX6C, ↓EPHA2, ↓FOXO1, ...all 24
CpG ODN 2006	chemical reagent	Activated	2.008	1.55E-01	↑CD274, ↑GBP1, ↑IL2RB, ↑LYN, ↑MVP, ↓MX1, ↑NOTC... ...all 11
1-palmitoyl-2-(5-oxovaleroyl)-sn-glyce	chemical reagent	Activated	2.000	1.76E-02	↓MYOCD, ↑SNAI1, ↑TGFB1, ↑TWIST1 ...all 4
Mrgpra2a (includes others)	G-protein coupled receptor	Activated	2.000	4.00E-02	↑CD274, ↑CXCR2, ↑GRINA, ↑TNFRSF1B ...all 4
ITGAV	transmembrane receptor	Activated	2.000	9.85E-02	↑AMIGO2, ↑ITGA1, ↑SERPINE1, ↑TGFB1 ...all 4
niraparib	chemical drug	Activated	2.000	1.29E-02	↑ECM1, ↑GBP2, ↑RMRP, ↑TNFSF10 ...all 4
lurbinectedin	chemical drug	Activated	2.000	1.51E-03	↑JAG1, ↑KLF10, ↑NOTCH2, ↑SAT1 ...all 4
SPEN	transcription regulator	Activated	2.000	1.08E-01	↑RPL22L1, ↑RPL30, ↑RPL8, ↑RPS20 ...all 4
MAP2K3	kinase	Activated	2.000	1.43E-01	↑SERPINE1, ↑SGK1, ↑SLC5A5, ↑TNFSF10 ...all 4
M344	chemical reagent	Activated	2.000	3.21E-02	↓BACE1, ↑MAPT, ↑SERPINE1, ↑TGFB1 ...all 4

Figure S1: Ingenuity Pathway Analysis (IPA) of Bulk RNAseq data from ARHGAP18 KO compared to WT-Jeg3. A) Graphical representation of predicted interactions. Nearly all observed protein-protein interactions are separated by a grey line indicating "Interaction not predicted". Notable exceptions include RhoA, CDH1, and SMAD4 B) List of upstream regulator predicted to be involved due to expressional changes seen in the dataset. C) List of upstream regulator predicted to be involved due to expressional changes seen in the dataset.

Gene	Description	Comment	Citation
AUTS2	Activator Of Transcription And Developmental Regulator AUTS2	Part of the PRC1-like complex	https://pubmed.ncbi.nlm.nih.gov/25519132/
CARMIL2	Capping Protein Regulator And Myosin 1 Linker 2	Membrane bound actin capping protein	https://pubmed.ncbi.nlm.nih.gov/26578515/
CYP7B1	Cytochrome P450 Family 7 Subfamily B Member 1	Metabolistm of steroids	https://pubmed.ncbi.nlm.nih.gov/10588945/
EMILIN2	Elastin Microfibril Interfacer 2	Microfibrillar Protein	https://pubmed.ncbi.nlm.nih.gov/26945878/
JCAD	Junctional Cadherin 5 Associated Protein	Cell-to-cell junction protein regulating Hippo signaling	https://pubmed.ncbi.nlm.nih.gov/29794114/
AMOT	Angiomotin	Repressor of YAP1	https://pubmed.ncbi.nlm.nih.gov/21205866/
PTPRG	Protein Tyrosine Phosphatase Receptor Type G	Growth factor receptor	https://pubmed.ncbi.nlm.nih.gov/16263724/
WNT11	Wnt Family Member 11	WNT gene family member	https://pubmed.ncbi.nlm.nih.gov/11712081/
HDAC7	Histone Deacetylase 7	Regulates activity of FOXP3	https://pubmed.ncbi.nlm.nih.gov/17360565/
TPM1	Tropomyosin 1	Actin binding protein which regulates myosin binding activity	https://pubmed.ncbi.nlm.nih.gov/38971309/
WWC2	WW And C2 Domain Containing 2	Hippo Pathway component. Regulates phosphorylation of YAP1	https://pubmed.ncbi.nlm.nih.gov/24682284
CD24	Precurer protein to a sialoglycoprotein	Expressed on cell exterior	https://pubmed.ncbi.nlm.nih.gov/32790899/
WDR62	WD Repeat Domain 62	MAPK binding protein 1 paralog	https://pubmed.ncbi.nlm.nih.gov/19910486/
AQP1	Aquaporin 1	Water channel membrane protein	https://pubmed.ncbi.nlm.nih.gov/9177353/
SH3D21	SH3 Domain Containing 21	EGFR signaling	https://pubmed.ncbi.nlm.nih.gov/20029029
KITLG	KIT Ligand	Stimulates phosphorylation of PIK3 and activation of AKT1 with activation of MAP kinases	https://pubmed.ncbi.nlm.nih.gov/32463597/
SFRP1	Secreted Frizzled Related Protein 1	WNT Antagonist	https://pubmed.ncbi.nlm.nih.gov/14871816/
EPHA2	Ephrin Receptor A2	Inhibition of the ERK1/ERK2 (MAPK3/MAPK1) signaling pathway	https://pubmed.ncbi.nlm.nih.gov/12400011/
BMPR1A	Bone Morphogenetic Protein Receptor Type 1A	Transmembrane serine/threonine kinase invovled in PI3K/AKT signaling	https://pubmed.ncbi.nlm.nih.gov/25110865/
GDF6	Growth Differentiation Factor 6	Secreted ligand of the TGF-beta	https://pubmed.ncbi.nlm.nih.gov/23307924/
BMP4	Bone Morphogenetic Protein 4	Ligand activating ERK/MAP kinase, PI3K/Akt	https://pubmed.ncbi.nlm.nih.gov/31363885
F3	Coagulation Factor III	Cell surface glycoprotein initiates the blood coagulation cascade	https://pubmed.ncbi.nlm.nih.gov/25535411/
ID1	Inhibitor of DNA Binding 1	Interferes with DNA binding of Transcription factors	https://pubmed.ncbi.nlm.nih.gov/10537105/
CXADR	CXADR Ig-Like Cell Adhesion Molecule	Activates PI3-kinase and MAP kinases	https://pubmed.ncbi.nlm.nih.gov/38146657
LOXL2	Lysyl Oxidase Like 2	Transcription corepressor	https://pubmed.ncbi.nlm.nih.gov/27735137/
PODXL	Podocalyxin Like	Ezrin Binding. MAPK / PI3K	https://pubmed.ncbi.nlm.nih.gov/17616675
ANXA3	Annexin A3	Negative regulator of the mitogen-activated protein kinase (MAPK) pathway, promoting the targeting of EGFR to lysosomes for degradation	https://pubmed.ncbi.nlm.nih.gov/22797061/
L1CAM	L1 Cell Adhesion Molecule	Regulates cell adhesion and the generation of transmembrane signals	https://pubmed.ncbi.nlm.nih.gov/22796939/
ITGB5	Integrin Subunit Beta 5	Integrin regulated by Hippo signaling and YAP/TAZ	https://pubmed.ncbi.nlm.nih.gov/28504269/
SSH2	Slingshot Protein Phosphatase 2	Cofillin Hippo	https://pubmed.ncbi.nlm.nih.gov/25864508/
P2RY6	Pyrimidinergic Receptor P2Y6	G-Protein Coupled receptor activated by extracellular nucleotides	https://pubmed.ncbi.nlm.nih.gov/39322240/
SPN	Sialophorin (CD43)	ERM binding protein	https://pubmed.ncbi.nlm.nih.gov/10385528/
ITGB4	Integrin Subunit Beta 4	Integrin regulated by Hippo signaling and YAP/TAZ	https://pubmed.ncbi.nlm.nih.gov/28504269/
SEMA3D	Semaphorin 3D	Activates Hippo Signaling	https://pubmed.ncbi.nlm.nih.gov/35613278/
FOXP1	Forkhead Box P1	Direct YAP/TAZ binding protein	https://pubmed.ncbi.nlm.nih.gov/24525530/
TNS1	Tensin1	Focal adhesion protein. Activates YAP.	https://pubmed.ncbi.nlm.nih.gov/38297127/
BAMBI	BMP and activin membrane-bound inhibitor	Target gene of Bmp-4 signaling.	https://pubmed.ncbi.nlm.nih.gov/11165491/
PREX1	Phosphatidylinositol-3,4,5-Trisphosphate Dependent Rac Exchange Factor 1	Rac1 GEF stimulated by PI3K/ ERK signaling	https://pubmed.ncbi.nlm.nih.gov/17308088/
FAT2	FAT Atypical Cadherin 2	Member of the cadherin superfamily. Supresses YAP activation	https://pubmed.ncbi.nlm.nih.gov/29985391/
APOE	Apolipoprotein E	Activates MAP3K12 and a non-canonical MAPK signal transduction pathway	https://pubmed.ncbi.nlm.nih.gov/28111074
PRKG1	Protein Kinase CGMP-Dependent 1	Activates MAPK Kinase (MEK)	https://pubmed.ncbi.nlm.nih.gov/10567406/
HMOX1	Heme Oxygenase 1	Catalyzes the oxidative cleavage of heme	https://pubmed.ncbi.nlm.nih.gov/11121422/
SPNS2	SPNS Lysolipid Transporter 2, Sphingosine-1-Phosphate	Lipid transporter of sphingosine 1-phosphate	https://pubmed.ncbi.nlm.nih.gov/19074308/
EMP2	Epithelial Membrane Protein 2	Regulates integrins	https://pubmed.ncbi.nlm.nih.gov/16216233
FGFR4	Fibroblast Growth Factor Receptor 4	Cell surface receptor that mediates activation of MAP kinase signaling pathway	https://pubmed.ncbi.nlm.nih.gov/21203561/
NRP2	Neuropilin-2	Transmembrane receptor. Binds Semaporin 3	https://pubmed.ncbi.nlm.nih.gov/28843905/
WNT4	Wnt Family Member 4	Wnt Family Member 4	https://pubmed.ncbi.nlm.nih.gov/24964196/
ITGA2	Integrin Subunit Alpha 2	Integrin regulated by Hippo signaling and YAP/TAZ	https://pubmed.ncbi.nlm.nih.gov/28504269/
MYOCD	Myocardin	Regulated by YAP/TAZ	https://pubmed.ncbi.nlm.nih.gov/37927241/
CCBE1	Collagen And Calcium Binding EGF Domains 1	Involved in binding to components of the extracellular matrix	https://pubmed.ncbi.nlm.nih.gov/21778431/

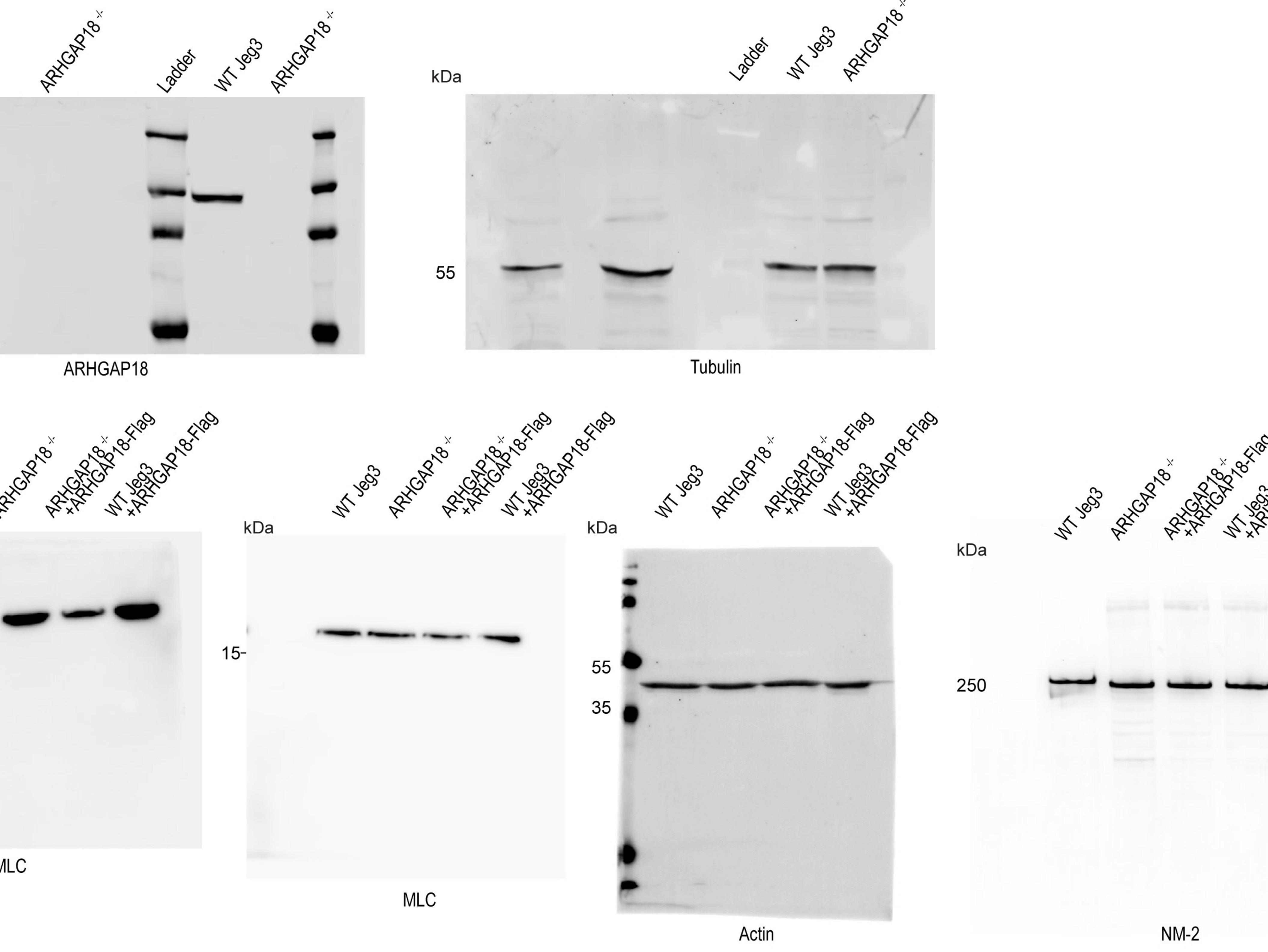
ARHGAP18 -/-
Ladder
WT Jeg3
ARHGAP18 -/-
ARHGAP18
kDa
Ladder
WT Jeg3
ARHGAP18 -/-
55
Tubulin
ARHGAP18 -/-
ARHGAP18 -/- +ARHGAP18-Flag
WT Jeg3 +ARHGAP18-Flag
MLC
kDa
WT Jeg3
ARHGAP18 -/-
ARHGAP18 -/- +ARHGAP18-Flag
WT Jeg3 +ARHGAP18-Flag
15
MLC
kDa
WT Jeg3
ARHGAP18 -/-
ARHGAP18 -/- +ARHGAP18-Flag
WT Jeg3 +ARHGAP18-Flag
55
35
Actin
kDa
WT Jeg3
ARHGAP18 -/-
ARHGAP18 -/- +ARHGAP18-Flag
250
NM-2

Fig. 5A

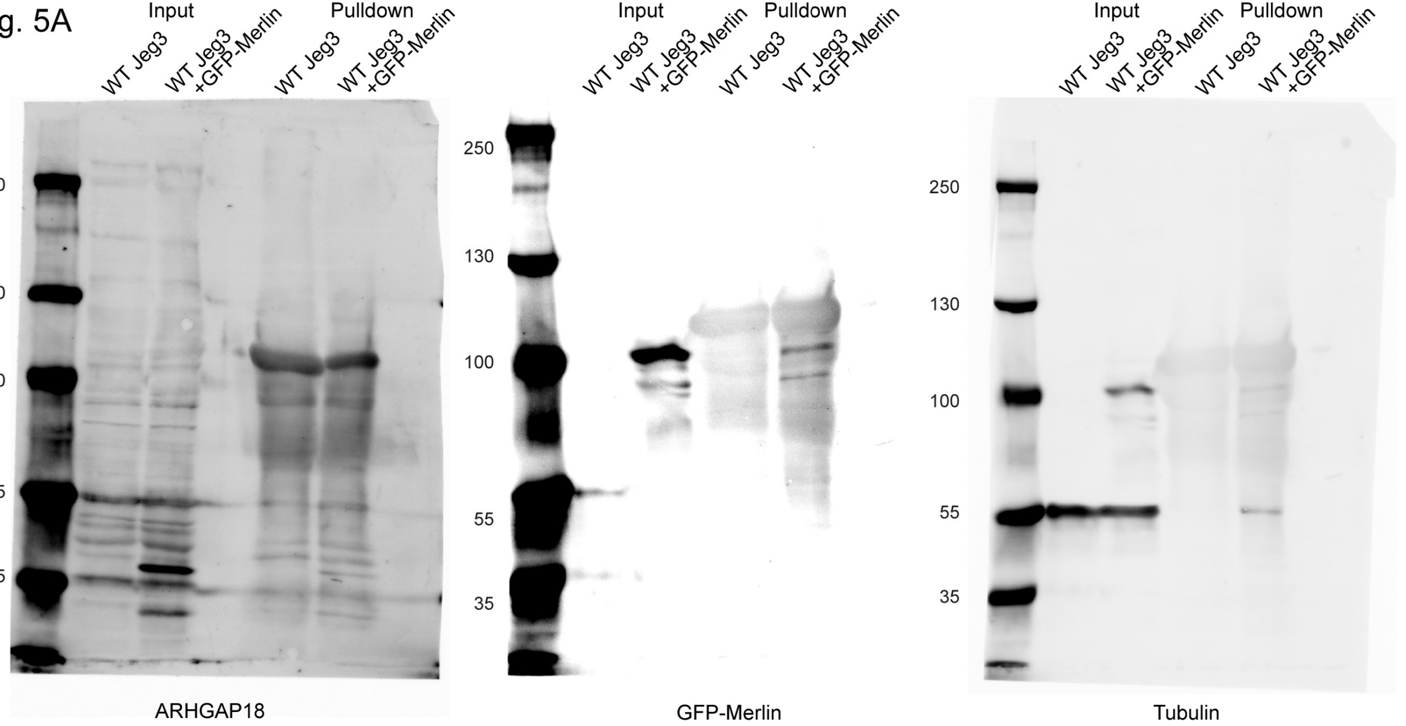

Fig. 5E

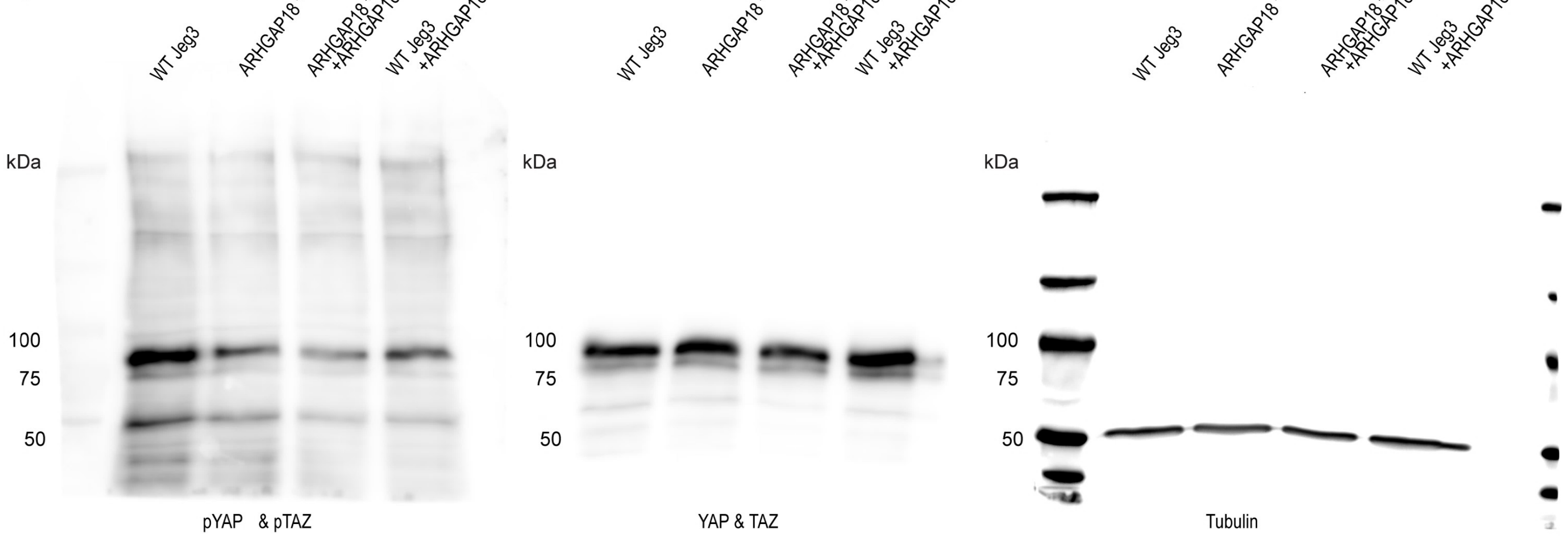

Fig. 5G

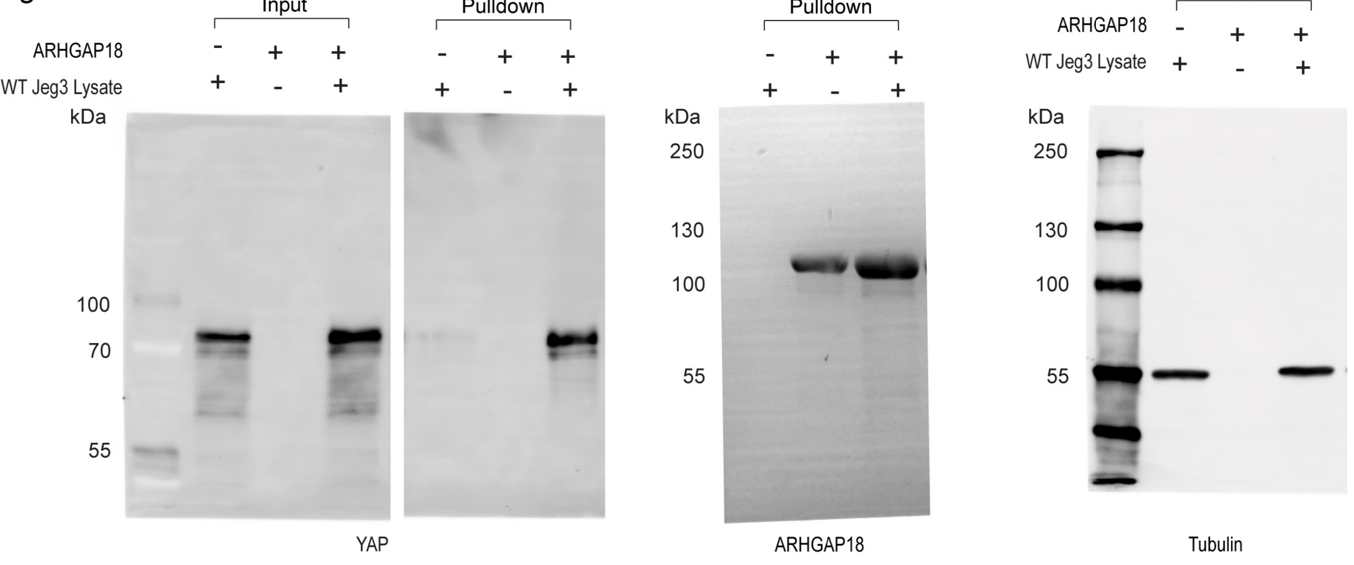

Supplement: Supplementary file 1 [file mbc-37-ar75-s001.pdf]
